# An In-Depth Exploration of Unconventional Machining Techniques for INCONEL^®^ Alloys

**DOI:** 10.3390/ma17051197

**Published:** 2024-03-04

**Authors:** André F. V. Pedroso, Naiara P. V. Sebbe, Francisco J. G. Silva, Raul D. S. G. Campilho, Rita C. M. Sales-Contini, Rui P. Martinho, Rafaela B. Casais

**Affiliations:** 1 CIDEM, ISEP, Polytechnic of Porto, Rua Dr. António Bernardino de Almeida, 4249-015 Porto, Portugal; afvpe@isep.ipp.pt (A.F.V.P.); napvs@isep.ipp.pt (N.P.V.S.); rds@isep.ipp.pt (R.D.S.G.C.); or rita.sales@fatec.sp.gov.br (R.C.M.S.-C.); rpm@isep.ipp.pt (R.P.M.); rbc@isep.ipp.pt (R.B.C.); 2 LAETA-INEGI, Associate Laboratory for Energy, Transports and Aerospace, Rua Dr. Roberto Frias 400, 4200-465 Porto, Portugal; 3 Technological College of São José dos Campos, Centro Paula Souza, Avenida Cesare Mansueto Giulio Lattes, 1350 Distrito Eugênio de Melo, São José dos Campos 12247-014, Brazil

**Keywords:** INCONEL^®^ 625, INCONEL^®^ 718, non-conventional processes, rough machining processes, surface finish processes

## Abstract

Build-up-edge (BUE), high-temperature machining and tool wear (TW) are some of the problems associated with difficult-to-machine materials for high-temperature applications, contributing significantly to high-cost manufacturing and poor tool life (TL) management. A detailed review of non-traditional machining processes that ease the machinability of INCONEL^®^, decrease manufacturing costs and suppress assembly complications is thus of paramount significance. Progress taken within the field of INCONEL^®^ non-conventional processes from 2016 to 2023, the most recent solutions found in the industry, and the prospects from researchers have been analysed and presented. In ensuing research, it was quickly noticeable that some techniques are yet to be intensely exploited. Non-conventional INCONEL^®^ machining processes have characteristics that can effectively increase the mechanical properties of the produced components without tool-workpiece contact, posing significant advantages over traditional manufacturing.

## 1. Introduction

**Figure 1:**
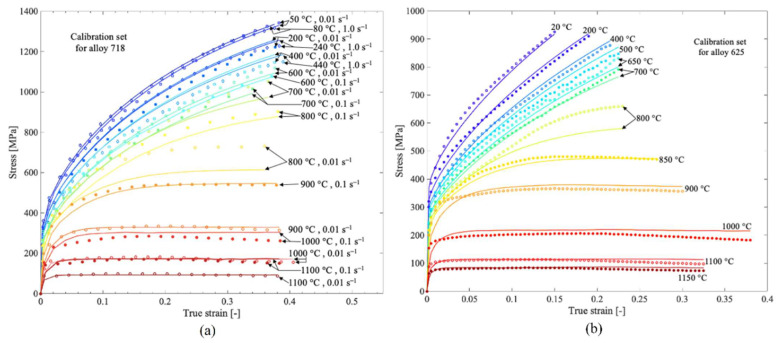
Measured true-stress–strain (*σ_tr_*–*ε_tr_*) curves (discrete points) for (**a**) INCONEL^®^ 718 and (**b**) INCONEL^®^ 625. Corresponding computed results (solid lines) from the material model after calibration. The tests were performed with a nominal $\dot{\varepsilon }$ = 0.01 Hz for INCONEL^®^ 625, while INCONEL^®^ 718 was tested with 0.01 < $\dot{\varepsilon }$ < 1 Hz [8].

A suitable material choice for high-temperature requirements is the INCONEL^®^ alloy, with a typical volumetric mass density (*ρ*) of 8908 kg/m^3^ [1]. Being one of the most representative-strengthening Ni-superalloys, INCONEL^®^ 718 has a face-centred cubic (FCC) γ matrix, having as main precipitations the gamma prime phase (γ’, primary strengthening phase in Ni-based superalloy with an ordered L_12_ FCC crystal structure [2]) Ni_3_(Al, Ti, Nb) and the double gamma phase (γ”, the main strengthening phase in INCONEL^®^ 718 with ordered D022 Body-Centred Tetragonal, BCT, structure [2]) Ni_3_Nb [3,4]. On the other hand, INCONEL^®^ 625 has a different solid solution hardening with Nb and Mo within the Ni-Cr matrix [5]. Figure 1 presents the different elastoplastic behaviours between INCONEL^®^ 718 and 625. Due to the chemical composition, Ni-based superalloys have resistance to oxidation, caustic and high-purity water corrosion, creep and stress-corrosion cracking (SCC) [6]. A crucial physical property for extremely high-temperature environments is low thermal conductivity (*k*) [7].

**Figure 2:**
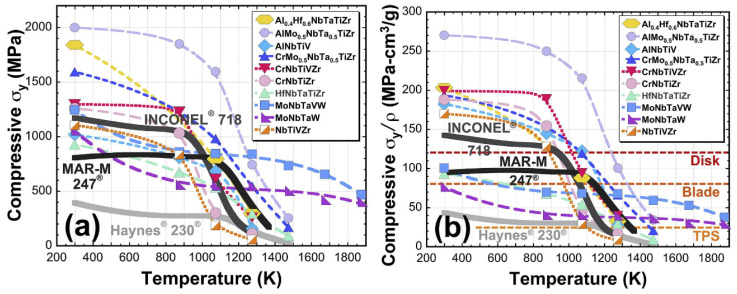
(**a**) *σ*_yc_ and (**b**) *σ*_yc_/*ρ* dependent on *T*. Typical *σ*_yc_/*ρ* requirements for thermal protection sheet, turbine blades and disks are shown in [9].

Figure 2 displays the temperature (*T*) dependence of compressive yield strength (*σ*_yc_, Figure 2a) and the specific compressive strength (*σ*_yc_/*ρ*, Figure 2b) of INCONEL^®^ 718 and the comparison with high-entropy alloys and two other Ni-based alloys.

**Figure 3:**
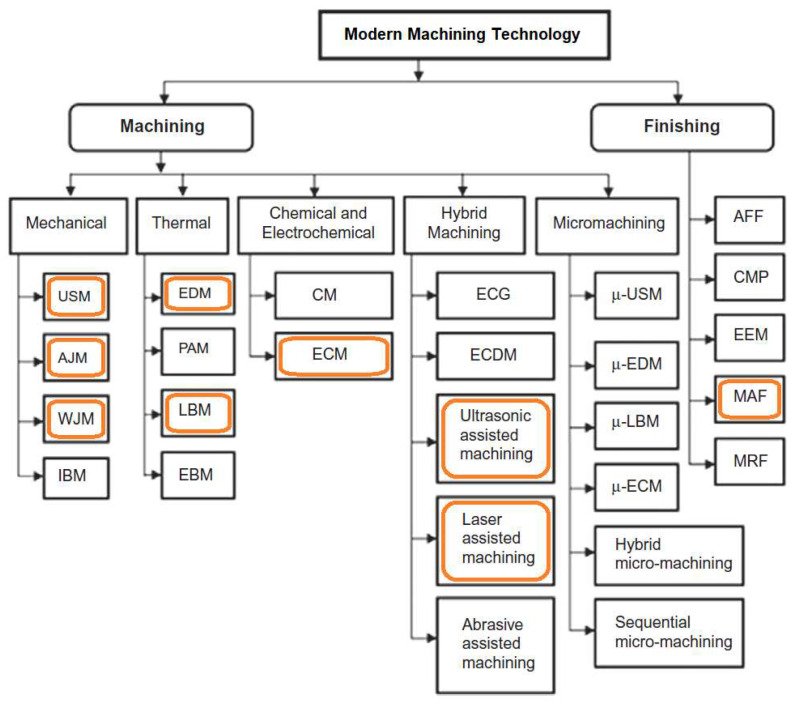
Classification of modern machining technologies (adapted from [28]).

However, this property makes INCONEL^®^ alloys a hard-to-machine-metal [10] and difficult-to-metal-shape substance, influencing heat distribution during machining and impacting the surface quality when employing conventional chip-start cutting machining and surface treatment techniques [11]. Conventional manufacturing (CM) and the instantaneous work hardening in the chip formation region due to the creation of a surface with several porosities make INCONEL^®^ alloys prone to reduced low-cycle fatigue (LCF) strength [12] and corrosion, leading to faster oxidisation by ammonium chloride (NH_4_Cl) [13], H_2_ embrittlement in electrolysis processes environments [14,15] and sulphuration corrosion failure [16]. Both work hardening and H_2_ embrittlement induce increased hardness, which, in turn, induces a decrease in toughness and fatigue resistance. Moreover, the porosity induced by some INCONEL^®^ alloy processing also contributes to a significant decrease in toughness and fatigue resistance, facilitating crack generation and propagation. Yin et al. [17] found that applying a WC-10Co-4Cr coating through a supersonic flame of high-velocity oxygen fuel (HVOF) on INCONEL^®^ 690 alloy surface improved the wear resistance, decreasing the corrosion rate to seawater. Zhang et al. [18] investigated the subgrain segregation between the dendrite core and inter-dendritic region of INCONEL^®^ 625 manufactured by wire arc additive manufacturing (WAAM), claiming N(Nb, Ti) precipitates in the inter-dendritic region significantly reinforce the Ni-based superalloy corrosion resistance, fabricated by additive manufacturing (AM). Soundararajan et al. [19] applied the electrochemical nanoindentation (ECNI) method to probe the nanomechanical properties of INCONEL^®^ 625 under an H_2_ environment to predict and prevent embrittlement. Rodriguez et al. [20] studied the corrosion behaviour of INCONEL^®^ 625 and 718 in subcritical, supercritical and ultra-supercritical water exposure as a function of *T* and time (Δ*t*). Short-term exposure tests promoted the breakdown of the native oxidate film of INCONEL^®^, and mixtures of oxides containing Ni, Fe, Cr and Nb formed on the surface and an Fe-rich oxide layer on an INCONEL^®^ 718 surface that prevents mass loss.Traditional manufacturing also instigates residual tensile and compressive stresses into the materials’ machined surface, leading to plastic deformation in the INCONEL^®^ wrought-stock or the tool. This causes patterns in the flank wear (*VB*_max_) and, consequently, BUE formation, thus resulting in premature tool failure [21,22]. Hence, these considerations have raised the search for innovative manufacturing methods [23] for these hard-metal alloys. Some of the addressed processes are highlighted in Figure 3. For instance, INCONEL^®^ 718 can be additively manufactured (AMed) to produce components with complex shapes. Since it relies on a γ” hardening phase Ni_3_Nb (D022) and a low amount of γ′ forming elements, namely FCC Ni_3_(Ti, Al, Nb) (L12), it has excellent weldability (using electron beam welding, EBW, as an example [24]) and weld-repair ability compared to other γ′-strengthened alloys [2,25,26].Wang et al. [27] examined the microstructural evolution and creep characteristics of INCONEL^®^ 718 alloy subjected to varying temperatures and stresses. 

**Figure 4:**
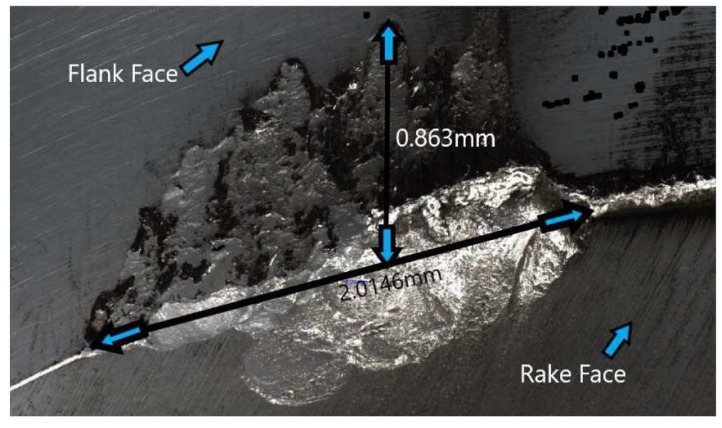
Image of the width and height of the wear scar on insert A (SiAlON grade 1). The image was captured on an Alicona infinite-focus microscope [36].

The study revealed that, as creep advanced, the stability of the γ” phase diminished, leading to its transformation in the δ phase. Additionally, an acceleration in the transformation from γ” to δ was observed with increasing temperature and stress. A model for creep fracture was formulated to enhance the prediction of these phenomena. In the AM field, Wang et al. [29] researched creep properties and microstructures of INCONEL^®^ 718 fabricated using laser-powder bed fusion (LPBF), varying with the height of the structure, conducted at 650 °C/800 MPa. The authors determined that the bottom specimens have longer creep life than the middle and top specimens due to the lower dislocation density and the higher Taylor factor, which cause a lower creep rate and grain boundary (GB) volume fraction, which reduces the void nucleation. Kim et al. [30] investigated the high-temperature tensile stress and high cycle fatigue properties of INCONEL^®^ 625 fabricated through LPBF and compared the results with the conventional wrought INCONEL^®^ 625. Yield and tensile strengths (*σ*_y_ and *σ*_u_, respectively) and the elongation at fracture (*ε*_u_) were similar at room *T*. For *T* = 650 °C AMed, INCONEL^®^ 625 has a significantly lower *ε*_u_ and high-cycle fatigue limit of 500 MPa @ 10^7^ cycles compared to the wrought INCONEL^®^ 625 with 575 MPa. The authors suggest that the high S content (33 ppm) in the AMed INCONEL^®^ 625 might be the reason for the lower fatigue endurance. From a pure manufacturing feasibility point of view, it is imperative to explore non-traditional manufacturing processes since CM tends to bring significant challenges in INCONEL^®^ machining, such as surface integrity, surface roughness (SR) and considerable TW rate. Kurniawan et al. [31] investigated the machinability of INCONEL^®^ 713C since it is a difficult-to-cut material and causes severe TW with BUE formation and catastrophic fracture. The commercial WC TiAlN-coated tool [32,33] used in the experiments suffered mainly from erosion on the cutting edge due to diffusion wear after the abrasive wear when cutting INCONEL^®^ 713C. This type of coating, TiAlN, and its variants TiN [34], TiAlYN [35] and TiAlVN, have been experimented with in INCONEL^®^ 718. Osmond et al. [36] described the mechanisms involved in chip formation and wear for SiAlON-based ceramics and silicon carbide whisker-reinforced alumina (WRA, Figure 4) round inserts during the turning process of solution-annealed INCONEL^®^ 718, utilizing a cutting fluid with an oil-concentration of 10%.

Using SiAlON turning inserts at a cutting speed (*v*_c_) of 300 mm/min delivered the best results in terms of tool *VB*_max_, as in ISO 3685:1993(E) [37], total tool life (TL) and workpiece surface finish. Toubhans et al. [38] investigated the machinability of INCONEL^®^ 718, employing a circular carbide tool under finish turning conditions. Cutting forces (*F*_c_) [39] and evolution during TL were analysed, leading to the development of an original mechanistic *F*_c_ model. The new local formulation accurately predicted *F*_c_ evolution over various finishing parameters during the initial running-in and controlled wear phases. The fluctuation in the evolution of tool wear remains a challenge in developing thoroughly robust models. Pleta et al. [40] experimented with the trochoidal milling process on INCONEL^®^ 718 and optimisation for milling scenarios, where machining parameters are investigated as to how they relate to improving TL and *F*_c_ using the Taguchi method. The undeformed chip thickness (*h*_ch_) increased as TW increased up to the depth of the plastically deformed grains in the axial and radial orientations. Also, the rotational rate ($\dot{\theta }$) and nutational rate ($\dot{\varphi }$) have the most significant interactions with *F*_c_ and *VB*_max_, as in ISO 8688-2:1989(E) [41]. Suárez et al. [42] explored the influence of ultrasonic vibration-assisted milling (UVAM) on the surface integrity and fatigue life of INCONEL^®^ 718, comparing it with abrasive water-jet machining (AWJM), wire electro-discharge machining (WEDM) and conventional milling (CM). The *R*_a_ values were 0.8 μm for CM, 0.2 μm for UVAM, 1.6 μm for AWJM and 3.4 μm for WEDM. WEDM surfaces generally exhibited elevated tensile residual stresses and the lowest fatigue endurance (less than 40,000 cycles). In contrast, UVAM surfaces demonstrated superior fatigue performance (approximately 65,000 cycles) and the highest compressive residual stresses (CRS), thereby extending the machined surface fatigue life by 12% compared to CM. High-accuracy machining of any hard-to-cut metal is the most substantial advantage of non-conventional processes, regardless of the shape complexity, while not requiring contact between the tool and the wrought stock. For further information about INCONEL^®^ 718 and 625 alloys and all surrounding subjects, such as microstructure, machining problems and predictive mathematical models, we suggest Pedroso et al. [43] for better comprehension.

An insight into INCONEL^®^ alloys and the addressed non-conventional processes has been given in the presentation of the theoretical framework in Section 1, which represents an extension of the review by Pedroso et al. [44]. Section 2 delineates the methodology employed in this study, which is based on the systematic literature review (SLR) approach [45] aimed at summarising how the research was conducted. Section 3 reviews different non-conventional machining techniques, depicting evolutional trends and the remarks of the researcher’s work. Section 4 discusses findings derived from content analysis, and Section 5 succinctly summarises the findings and offers a brief outlook on INCONEL^®^ alloy machining simulation.

## 2. Materials and Methods

The research and information-compiling phases were carried out through the SLR approach since it is based on a systematic, method-driven and replicable approach [46,47]. The platform used for SLR was Dimensions.ai (Digital Science, London, UK), which is connected to all data from Scopus (Elsevier, Amsterdam, The Netherlands) through quality criteria by consulting the journal influence within the academic community by impact factor (IF). This article was accomplished by compiling information from 90 reports, 29 book chapters and 5 standards from 189 articles and 19 books researched. The procedure is explained below with the following topics.

1. Information was searched with the “INCONEL^®^”, “INCONEL^®^ 718” and “INCONEL^®^625” keywords to gather more broad information about those Ni-based alloys.
2. The keyword “non-conventional manufacturing” was added to the previous ones, which enabled seeking information that compared traditional to non-traditional manufacturing processes.
3. Thanks to some review articles that enumerated non-conventional processes, such as “additive manufacturing with traditional processes”, “electrochemical machining”, and “electrical discharge machining”, among others addressed within this paper, this word merging strategy, alongside the combination of the process name to the material, was a central factor in obtaining the desired information.
4. After collecting the articles, the journal’s influence was evaluated with its Web of Science score from 2021 (ignoring quartiles). All journals with an IF value of less than three were excluded, although rounding to the unit was allowed.
5. Analysis of the abstract and conclusions from the collected articles proceeded.

Knowledge from 2016 to 2023 about the non-traditional process, INCONEL^®^ 718 and 625, was compiled.

## 3. Literature Review

### 3.1. Rough Machining Processes

This section addresses non-conventional manufacturing processes that allow the removal of considerable material from the workpiece.

#### 3.1.1. Electrochemical Machining (ECM)

The ECM process relies on electrochemical reactions to remove material from a workpiece, involving an electrolyte solution that will anodically dissolve the metal [48]. In contrast, thanks to an electrical current, a cathode shapes the workpiece to produce the desired shape at the atomic level with excellent material removal rate (MRR) standards [49,50]. This process is widely used in industries where precision and accuracy are paramount. ECM is used to machine complex shapes, thin-walled parts, heat-sensitive material drills and shape delicate components that cannot be machined with traditional methods. A variant of ECM is wire electrochemical moulding (WECM), which uses a wire as the cathode for intricate machine shapes in hard and brittle materials. Electrochemical mill-grinding (ECMG) is a hybrid machining process that combines ECM and mechanical grinding to remove material from a workpiece, used to machine high-precision parts like moulds, dies and aerospace components [51]. Electrochemical drilling (ECD) is a process that uses electrochemical reactions to remove material from a workpiece. ECD is a well-suitable process for drilling small, deep and precise holes for aerospace components.

**Figure 5:**
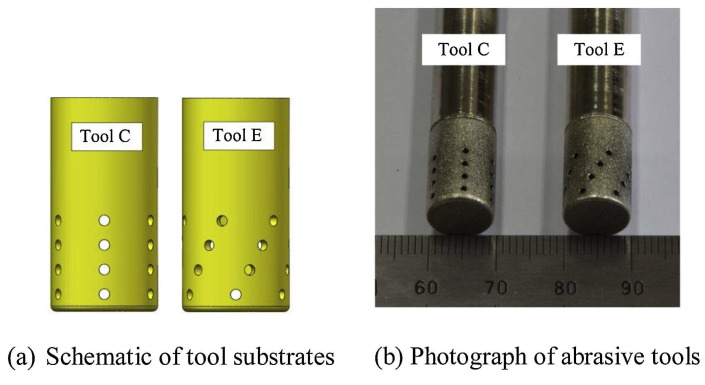
Different arrangements of tool-sidewall outlet holes: (**a**) schematic, (**b**) photo [53].

Manikanda et al. [52] studied multi-performance characteristics optimisation based on the Taguchi approach with grey relational analysis (TGRA) proposed for the ECD process on INCONEL^®^ 625, using an *L*_27_ orthogonal array. The TGRA optimisation proposed a single optimal solution for all the responses considering a feed rate (*f*) of 0.20 mm/min, flow rate (*Q*) of 0.60 L/min and electrolyte concentration of 25% to produce better machining characteristics. The optimal machining performance of ECD of INCONEL^®^ 625 at optimum machining conditions is evaluated with MRR = 0.1358 g/min, an arithmetic average of profile height deviation (*R*_a_) of 0.28 μm, overcut of 0.1811 mm, circularity error (*C*_I_) of 0.0631 mm and cylindricity error (*C*_Y_) of 0.0620 mm. Niu et al. [53] established that enhanced tool designs could be achieved during the preliminary stages of rough machining by modifying the arrangement and number of rows of tool-sidewall outlet holes for the ECMG process applied to INCONEL^®^ 718. Four tools with varying numbers of rows of tool-sidewall outlet holes were designed. The test results showed that an abrasive tool with four rows of tool-sidewall outlet holes could obtain a higher maximum *f*. Experimental results on machining a slot with this tool indicate that the MRR increases under a higher applied voltage (*U*), electrolyte pressure and *f*. In rough machining, the average sidewall flatness and the sidewall *R*_a_ obtained with the original tool are 549.6 μm and 2.509 μm, respectively. Comparing the results with the ones of the new tool (Figure 5), average sidewall flatness and the sidewall *R*_a_ are, respectively, 340.5 μm and 1.65 μm. The new tool is also applied for finish machining, producing an average sidewall flatness and the sidewall *R*_a_ of 69.5 μm and 0.648 μm, respectively.

**Figure 6:**
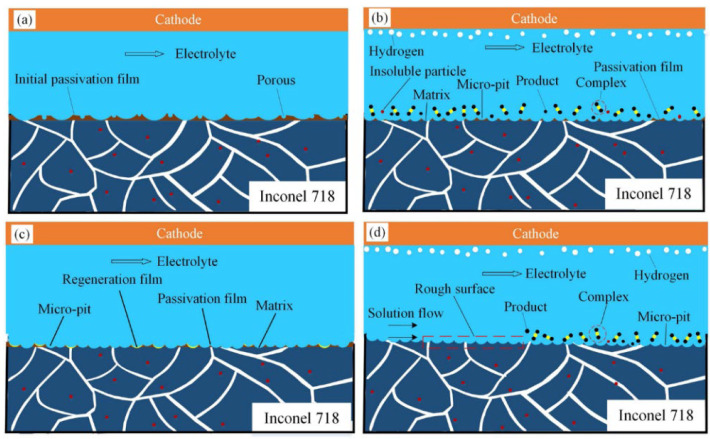
Schematic model of the electrochemical machining (ECM) behaviour of INCONEL^®^ 718 in C_6_H_5_K_3_O_7_ solution, (**a**) passivating film with a thin and loose porous structure, (**b**) few electrolytic products are formed where the passivating film is broken, and INCONEL^®^ 718 particles are gradually exposed to the electrolyte. A new passivation is regenerated during pulse-off time (*T*_off_), (**c**) passivation film and micro-pitting caused by C_6_H_5_K_3_O_7_ solution. (**d**) Elimination of electrolytic products, which stabilises the pulse ECM dissolution process and improves INCONEL^®^ 718 surface quality [54].

Wang et al. [54] evaluated the ECM of INCONEL^®^ 718 in potassium citrate (C_6_H_5_K_3_O_7_) electrolyte and clarified how the bulk material is removed, depicted in Figure 6. A comparison between C_6_H_5_K_3_O_7_ and NaNO_3_ solutions was made to verify the aftermath on the surface of the INCONEL^®^ 718 workpiece. Citric acid (Cit^3−^ or C_6_H_5_O_7_^3−^) ions substantially reduce the formation of flocculent and insoluble by-products in the machining area. The passivating film developed on the surface of INCONEL^®^ 718 in the C_6_H_5_K_3_O_7_ solution exhibited a loose and porous structure, distinct from that in the NaNO_3_ solution. The corrosion resistance of INCONEL^®^ 718 in the C_6_H_5_K_3_O_7_ solution was lower than in NaNO_3_ due to the aggressive nature of Cit^3−^. Additionally, a micro-pit structure was observed on the surface of INCONEL^®^ 718 after ECM in the former solution. The uniformity and scale of the micro-pit system dispersed on the machined surface improved with increased current density (*J*), thereby enhancing the surface quality of INCONEL^®^ 718.

**Figure 7:**
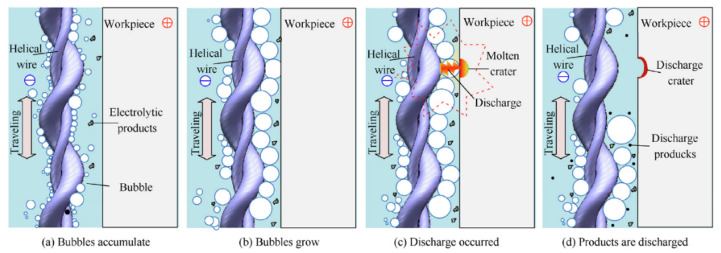
Principles of helical wire electrochemical discharge machining (HWECDM). (**a**) Anode reaction follows as M—ne^−^ → M^n+^, whereas cathode reaction follows as 2HOCH_2_CH_2_OH + 2e^−^ → 2HOCH_2_CH_2_O^−^ + H_2_↑. (**b**) The electrical conductivity of the working medium between the electrodes diminishes, resulting in an increase in the resistance of the electrolyte and an elevation of the electrical potential gradient between the electrodes. (**c**) A discharge channel forms at a protruding point of the helical wire electrode, leading to material removal from the workpiece. (**d**) The by-products of electrochemical machining (ECM) and electrical discharge machining (EDM) are expelled from the machining gap due to the combined effects of the explosive force from periodic electrical discharges and the axial movement of the helical wire electrode [55].

Kong et al. [55] observed that the intricate transport of electrolytic products in the confined machining gap poses challenges to machining large-thickness workpieces. A novel hybrid machining technique, helical wire electrochemical discharge machining (HWECDM), is introduced, combining the features of WECM and WEDM. This technique is applied to INCONEL^®^ 718 in a low-conductivity salt–glycol solution, as depicted in Figure 7. In the experiments, the minimum *R*_a_ and standard deviation (SD) values of the slit sidewall are 0.12 μm and 5 μm, respectively. The presence of discharges enhanced electrolytic product transport significantly, ensuring high machining efficiency, with a maximum value of *f* = 7 μm/s achieved for helical wire electrochemical discharge machining. It was demonstrated that HWECDM is a promising technique for machining large-thickness hard metal materials.

**Figure 8:**
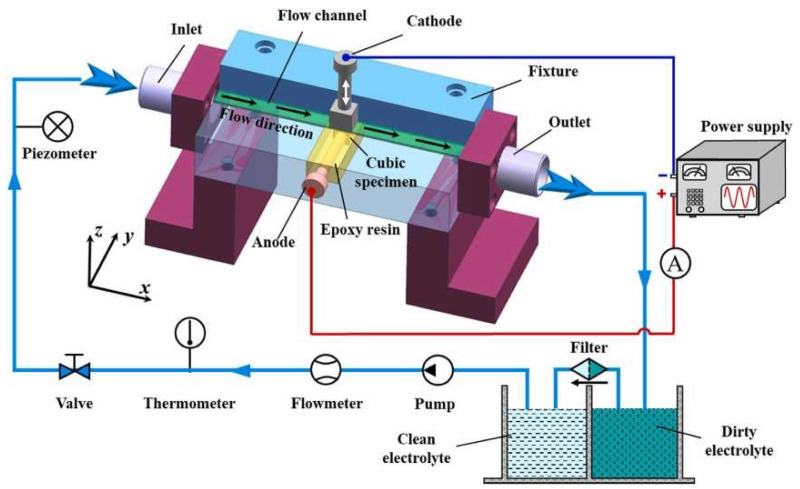
A 3D schematic of the experimental electrochemical machining (ECM) proceeding [56].

Ren et al. [56] conducted electrochemical tests and ECM on the modified phosphorus and boron-doped INCONEL^®^ 718 in a NaNO_3_ solution to examine its electrochemical dissolution behaviour. Figure 8 demonstrates how the experimental apparatus was mounted. Modified INCONEL^®^ 718 had significant active, passive and trans-passive regions in NaNO_3_ solution and is less corrosion-resistant than standard INCONEL^®^ 718 because of the inhomogeneous grain size. In ECM, uneven bulges developed due to the discrepancy in corrosion rates between coarse and refined grains, leading to a rough surface. As *J* increased, the passivation film was removed, and the δ phase and grains underwent simultaneous electrochemical dissolution. A value of *R*_a_ = 0.355 μm was obtained when *J* = 160 A/cm^2^. Finally, dissolution models were proposed to characterise the dissolution behaviour of doped INCONEL^®^ 718 in NaNO_3_ solution.

#### 3.1.2. Electrical Discharge Machining (EDM)

**Figure 9:**
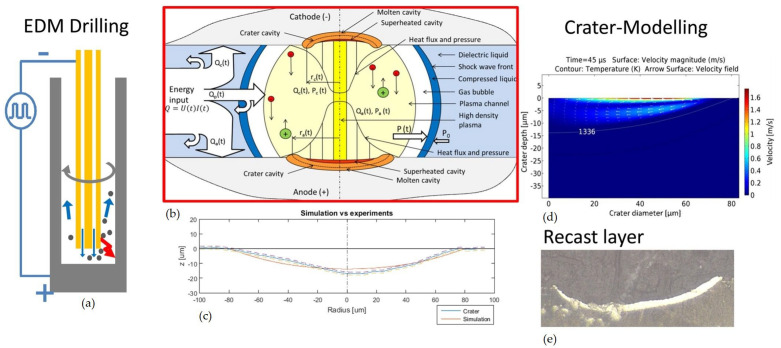
(**a**) EDM drilling schematic. (**b**) Examination of energy balance, crater formation, heat flux and pressure distribution during a single discharge in electrical discharge machining (EDM). (**c**) A comparison between simulated and measured crater shapes is presented as an illustration. (**d**) Investigation of the velocity field induced by the Marangoni effect [61] in the EDM melt pool simulation; (**e**) recast layer measurements, the craters are performed with the same set of parameters, the material is almost entirely ejected on the left and not ejected on the right (adapted from [58]).

EDM removes material from a wrought stock thanks to electrical discharges that develop high-energy plasma at *T* between 8000 °C and 20,000 °C, melting material and vaporising cavities from an electrode [57]. Figure 9, from Kliuev et al. [58], depicts a schematic of how EDM drilling is processed, the energy balance inherent to the manufacturing process and experimental versus numerical analysis-obtained results. The quality assessment for high precision and accuracy is measured by MRR and *R*_a_, parameters that are directly concerned by the heat-affected zone (HAZ) on metal and wire electrode meeting points [59]. WEDM is a variant of EDM that utilises a thin consumable wire as an electrode, which is very useful in producing intricate and delicate parts, such as medical components and electronic devices. The modulated short electric arc machining (MSEAM) process uses a modulated electric arc to remove material from a workpiece. It is used for high-speed machining (HSM) of difficult-to-machine materials. High-frequency electrical discharge-assisted milling (HF-EDAM) is a novel machining process investigated by Xu et al. [60], suitable for hard and brittle material, combining EDM and milling to remove material from a workpiece.

EDM and its variants are widely used in various industries, including aerospace, automotive, medical and electronics. Applications abound in these industries for which CM methods are ineffective [62] with hardened metals, such as INCONEL^®^ 718 and 625 [63], and complex geometries. Goyal [64] directed attention towards assessing the impact of process parameters on MRR and *R*_a_ in WEDM of INCONEL^®^ 625, involving using both standard and cryogenically treated zinc-coated wires. The design of experiments (DOE) method was employed, considering various and adjustable process parameters, including tool electrode, *J*, pulse-on time (*T*_on_), pulse-off time (*T*_off_), peak current (*I*_p_), wire feed and tension (WF and WT, respectively). Parameters such as servo voltage (*SV*), material thickness (*t*), wire diameter (*Ø*_w_) and dielectric flow rate are constant. A cryogenically cooled electrode provides better machining performance compared with ordinary wire. It has the maximum MRR value with the combination of these parameters: *I*_p_ = 12 A, *T*_on_ = 125 μs, *T*_off_ = 60 μs, WF = 8 m/min and WT = 9 N. *T*_on_ and *I*_p_ are found to be the most influential parameters for MRR and *R*_a_.

**Figure 10:**
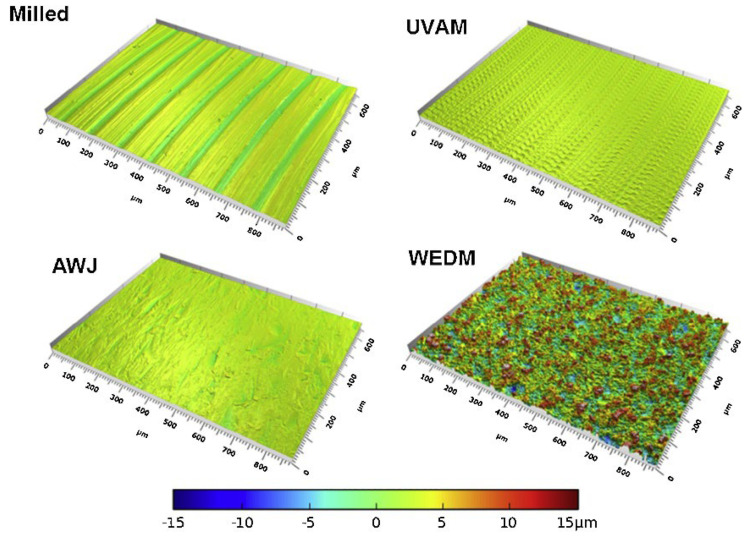
A 3D overview of the machined surface showing topography [42].

Suárez et al. [42] conducted a comparative analysis of alternative manufacturing processes, including AWJM, WEDM and UVAM, in comparison with CM, as illustrated in Figure 10. The study evaluated surface integrity, hardness, residual stress and fatigue strength resulting from these machining processes applied to the cutting of INCONEL^®^ 718. Regarding WEDM, the input parameters are *T*_on_ = 9 μs, *SV* = 65 V, voltage gap (*V*_g_) of 80 V, WT = 11.7 N, *I*_p_ = 6 A and WF = 4 mm/min. WEDM samples show *R*_a_ = 3.4 μm, with lower surface hardness. WEDM creates a state of high surface tensile stresses, which slightly decreases with depth. Results further indicate that the WEDM process significantly influences stress with a large depth below the surface. The WEDM sample surface had the lowest fatigue life from the four-point bending test. Overall, WEDM shows poorer results than other manufacturing processes addressed within the paper.

**Figure 11:**
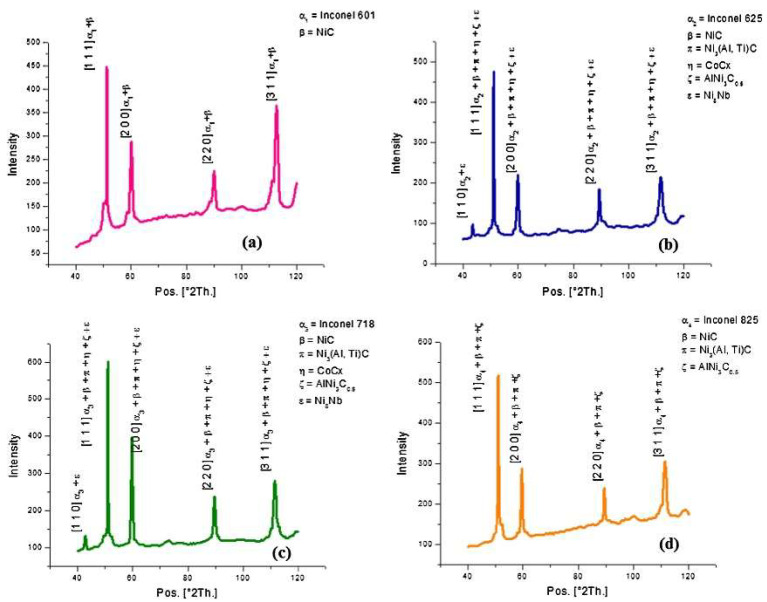
X-ray diffraction (XRD) spectra for the electrical discharge machining (EDM) treated work surface of (**a**) INCONEL^®^ 601, (**b**) INCONEL^®^ 625, (**c**) INCONEL^®^ 718 and (**d**) INCONEL^®^ 825 acquired under the parameter settings [*V*_g_ = 60 V, *I*_p_ = 5 A, *T*_on_ = 200 μs, *τ* = 70% and *F*_p_ = 0.3 bar] [65].

Rahul et al. [65] experimental EDM machining of INCONEL^®^ 718, 625, 825 and 601 had the main purpose of assessing the machinability of the four alloys, according to a five-factor/four-level *L*_16_ orthogonal array, applying a Taguchi method to determine optimal parameter settings. Variables such as *V*_g_, *I*_p_, *T*_on_, duty factor (*τ*) and flush pressure (*F*_p_) were considered. Machinability was assessed considering MRR, electrode wear rate, *R*_a_ and surface crack density (SCD). Also, XRD tests were considered to characterise the EDMed surface of the four INCONEL^®^ alloys (Figure 11). Only the INCONEL^®^ 718 and 625 alloys will be addressed in this state-of-the-art method. The results showed that for INCONEL^®^ 718 MRR = 1.1844–31.5995 mm^3^/min, and for INCONEL^®^ 625 MRR = 1.3389–29.3128 mm^3^/min. *V*_g_ = 70 V, *I*_p_ = 7 A, *T*_on_ = 500 μs, *τ* = 80% and *F*_p_ = 0.6 bar for INCONEL^®^ 718, and *V*_g_ = 80 V, *I*_p_ = 7 A, *T*_on_ = 200 μs, *τ* = 75%, and *F*_p_ = 0.6 bar for INCONEL^®^ 625 were the optimal parameters determined. *R*_a_ = 6–12.3667 μm for INCONEL^®^ 718 and *R*_a_ = 4.7–11.5333 μm for INCONEL^®^ 625 were the optimal SR values.

**Figure 12:**
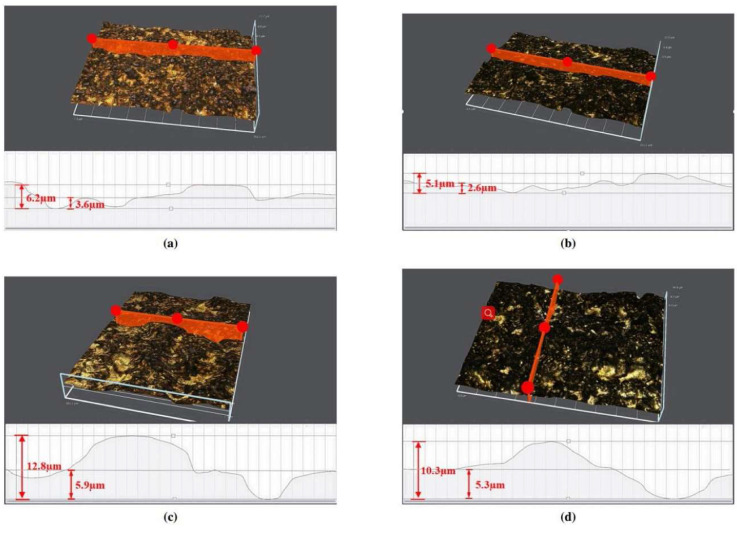
The 3D surface topographies of the electrical discharge machining (EDM)-treated surface under varying degrees of thermal deformation. Sample no. 4 with a deformation of 95.54 μm presented under (**a**) annular light and (**b**) coaxial light. Sample no. 33 with a deformation of 44.75 μm displayed under (**c**) annular light and (**d**) coaxial light [66].

Zhang et al. [66] elucidated the complex thermal deformation phenomena and their sources during the WEDM process of the thin-walled component made from INCONEL^®^ 718. A thermo–physical model was formulated to investigate thermal deformation by computing the temperature distribution of the workpiece and the resultant superficial residual stress. Then, experiments were conducted to investigate the effect of *T*_on_, *I*_p_, *F*_p_ and WF on the thermal deformation of thin-walled samples (Figure 12). Parameters such as *V*_g_, *T*_off_ and *f* were constant. Experimentally, thermal deformation was minimum within the typical parametric ranges of *T*_on_ = 16–18 μs, *F*_p_ = 0.5–0.7 MPa, WF = 0.25–0.29 m/s and *I*_p_ = 1–2 A. The generated residual stress on the surface in the machining process was dominated by the magnitude of thermal deformation and residual stress on the surface in the feed and wire directions.

**Figure 13:**
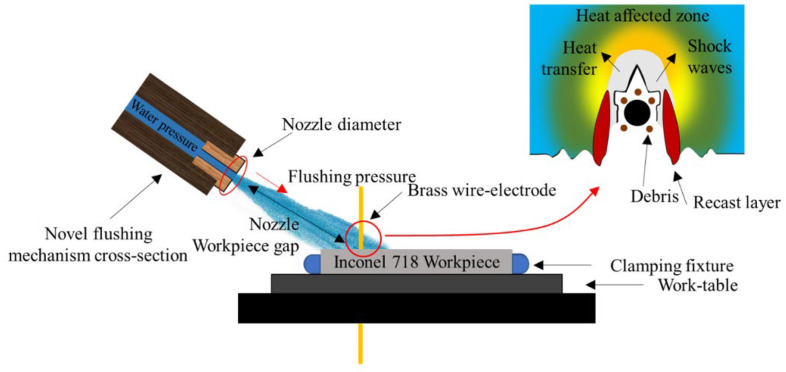
The innovative flushing mechanism implemented in the wire electro discharge machining (WEDM) by Farooq et al. [67].

Farooq et al. [67] studied the influence of *F*_p_ to accomplish debris exclusion when machining INCONEL^®^ 718, depicted in Figure 13, with WEDM, applying TGRA with an *L*_18_ array. The optimised parametric settings were *SV* = 50 V, *F*_p_ = 4 kg/cm^2^, nozzle diameter (*Ø*_N_) of 8 mm, and nozzle–workpiece distance (*W*_D_) of 10 mm.

**Figure 14:**
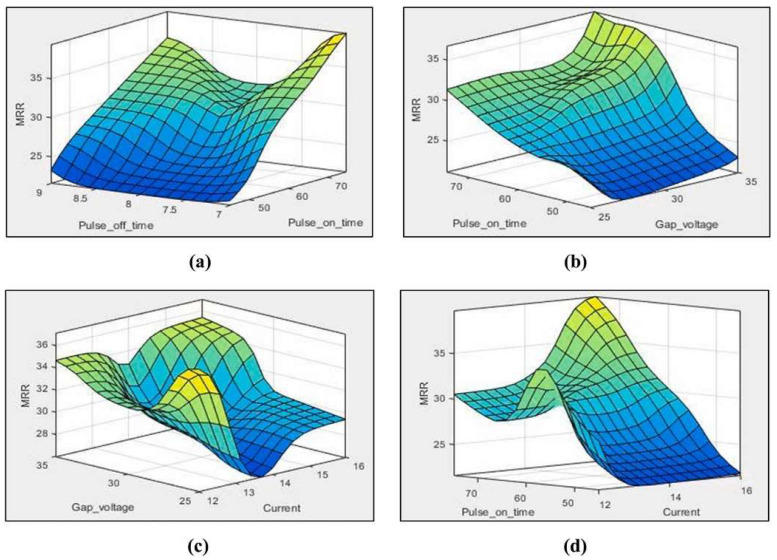
Interaction plots for material removal rate (MRR). (**a**) *T*_off_ vs. *T*_on_; (**b**) *T*_on_ vs. *V*_g_; (**c**) *V*_g_ vs. *I*_p_; (**d**) *T*_on_ vs. *I*_p_ [68].

Sharma et al. [68] determined the optimal machining parameters and the effect of the EDM process on MRR, *R*_a_ and TW. INCONEL^®^ 625 machining was conducted using a Cu-tool electrode in a kerosene-submerged medium. The optimum input parameters, *T*_on_, *T*_off_ and *V*_g_, were calculated to maximise MRR and minimise *R*_a_ and TW. An *L*_27_ orthogonal design was employed to perform the experiments. MRR increases as *T*_on_, *V*_g_ and *I*_p_ increase, and for the maximum value, the input parameters are *I*_p_ = 15–16 A and *T*_on_ = 69–75 μs, as seen in Figure 14. TW reaches its peak when *I*_p_, *T*_on_, and *T*_off_ are maximum because a substantial current and extensive pulse time generate considerable discharge energy, which causes an increase in TW. *T*_on_ and *T*_off_ greatly influenced *R*_a_; the lowest values were obtained when *V*_g_ = 25–75 V and *T*_on_ = 69–75 μs.

**Figure 15:**
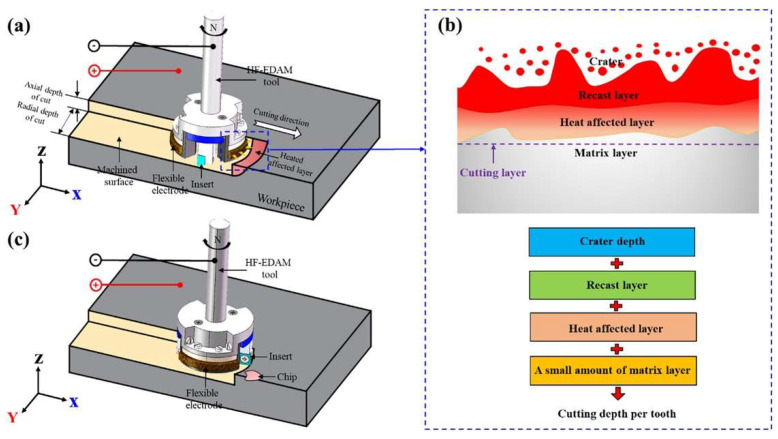
High-frequency electrical discharge-assisted milling (HF-EDAM) based on copper-beryllium bundle electrodes: (**a**) electrical discharge machining (EDM) process in HF-EDAM, (**b**) composition of the depth of cut (*a*_p_) after EDM and (**c**) milling process in HF-EDAM [60].

Kumar et al. [69] suggested a groundbreaking servo gap control mechanism utilizing actuator arm technology and magnetic levitation. The gap control mechanism is operated by balancing the repulsive electromagnetic and restoring forces (*F*_em_ and *F*_rs_, respectively). The machining feasibility of INCONEL^®^ 625 with maglev μ-EDM was examined. The specific energy consumption (SEC), while machining INCONEL^®^ 625 in EDM, was within 1.2245–1.6284 J/μg, MRR = 39–57 μg/min, TW = 4–9 μg/min and *R*_a_ = 0.899–1.057 μm. Also, the measured retraction speed was 1540.5 mm/min, which is 15× faster than traditional EDM, reaching typical retraction speeds of 100 mm/min. The authors claim the proposed technology might be an alternative to EDM servo gap control. Cu-Be alloys (UNS C17200, as established by ASTM B194–15 [70]) have combined properties of flexibility, wear resistance and high strength. HF-EDAM is a novel manufacturing process based on Cu-Be bundle electrodes developed for INCONEL^®^ 718. Xu et al. [60] purportedly investigated the hybrid EDM process by altering various machining parameters. In HF-EDAM experiments (Figure 15), Cu-Be bundle electrodes were observed to soften the workpiece’s surface during EDM while clearing the debris generated during milling. Due to the Cu-Be bundle electrode’s flexibility, the machined surface could be matched in real-time with the electrode, thereby effectively improving the discharge efficiency. Compared with CM, HF-EDAM significantly reduced *F*_c_ and the machined surface quality, demonstrating that this process is effective for the high-quality and efficient machining of INCONEL^®^ 718 machining.

#### 3.1.3. Hybrid Manufacturing Processes

This section presents a sequence of non-conventional manufacturing processes allied to traditional processes, which are called hybrid processes [71].

#### Additive Manufacturing (AM)

**Figure 16:**
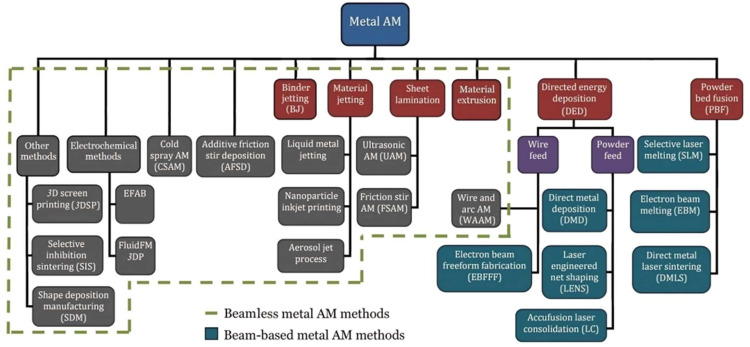
Classification diagram of metal additive manufacturing (AM) processes, highlighting beam-based and beamless processes (adapted from [79]).

The reader is advised that the review on AM intends to focus on the positive aspects of this process to traditional subtractive methods. AM is regarded as a sustainability-friendly and revolutionary technology, thanks to its significantly reduced production of debris/chipping [72] and the production of complex geometries and customised products with high precision and accuracy. Having the ArianeGroup case study as a brief example [25], the company successfully used INCONEL^®^ 718 and AM technologies to solve a technical challenge for the next generation of Ariane launchers related to a class 1 or mission-critical component. The ArianeGroup successfully implemented a simplified propulsion module, thanks to EOS GmbH technology, having an injector which is only one part instead of 248 elements. INCONEL^®^ 718 powder chemical composition was evaluated by ASTM B637–16 [73]. The diverse AM techniques can be classified based on feedstock type, energy source utilized, product build method, base material type and processing medium employed. The following widely used ones applied to INCONEL^®^ 718 alloys will be addressed in this paper: cold metal transfer (CMT), direct energy deposition (DED), LPBF and WAAM. Figure 16 portrays the classification of various metal AM processes available.

CMT relies on filler wire (electrode) short-circuit and precise wire control for material transfer, which belongs to the metal inert gas (MIG) technology [74,75]. DED [76] involves melting metal powder using a focused laser or electron beam to add material to an existing structure [77,78]. Two of the most known subprocesses of DED, classified as direct metal deposition (DMD), are direct laser deposition (DLD) and DEBD.

**Figure 17:**
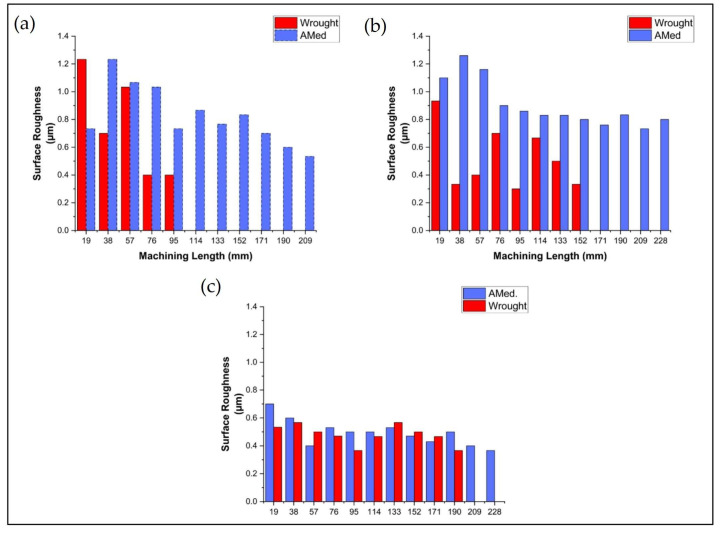
A comparison of *R*_a_ values concerning the machining length between additively manufactured (AMed) and wrought INCONEL^®^ 625 for different cutting environments: (**a**) dry; (**b**) electrostatic minimum quantity lubrication (EMQL); (**c**) CO_2_ (*l*) [88].

Avery et al. [80] also reported a somewhat homogeneous microstructure along with the thickness for INCONEL^®^ 625 specimens; however, the layer interfaces showed an even exceptional grain refinement, reaching submicron diameters on average. LPBF or selective laser melting (SLM) [81,82] uses a high-power laser to melt and fuse metal powder layer-by-layer to create a solid three-dimensional (3D) object [83], and the powder flow rate is measured according to ASTM B212–17 [84]. WAAM is a technique that uses an electric arc or plasma to melt down a metal welding wire [85], which is then deposited layer-by-layer to create a solid object, reaching deposition rates of 0.05–0.12 kg/min. However, it also generates undesirable postdeposition effects such as higher dilution, thermal distortion and a more significant HAZ [86]. Laves phase (an intermetallic compound with a stoichiometry of AB_2_ and is constructed when the atomic size ratio is between 1.05 and 1.67 [9]) also occurs in the WAAMed INCONEL^®^ 718 microstructure, needing a modified post-deposition heat treatment, which does not precipitate a δ phase (forms from decomposing γ″ with an orthorhombic structure Ni_3_Nb) [87]. Salvi et al. [88] carried out the economic analysis and CO_2_ emission assessment in the WAAM fabrication and machining of INCONEL^®^ 625 by evaluating TW, power consumption (*P*_in_) and *R*_a_ (Figure 17), comparing them to the traditional processes applied to a workpiece, maintaining the lubrication environments equal between all methods. One of the authors’ main findings was the increase of TL by about 191.55, 115.80 and 65.95% when machining AM-fabricated INCONEL^®^ 625, compared to milling the wrought stock, for dry, electrostatic minimum quantity lubrication (EMQL) and CO_2_ (*l*) machining environments, respectively. The continuous rise in tool wear rate and the ability of CO_2_ (*l*) to reduce the temperature in the cutting zone, thereby mitigating dissolution–diffusion tool wear, render the CO_2_ (*l*) cutting environment a suitable option.

**Figure 18:**
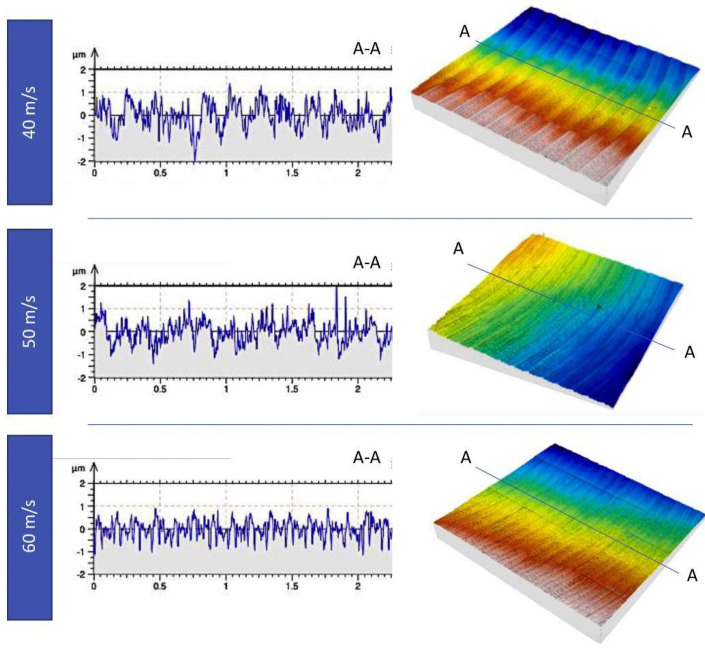
The roughness profiles and 3D topographies measured in the centre of the grooves (machining in building direction) [85].

Sharifitabar et al. [89] investigated and compared the corrosion resistance of WAAMed and a standard workpiece of INCONEL^®^ 625, showing that the former has 3.5% more resistance to corrosion. Alonso et al. [85] assessed the production of INCONEL^®^ 718 walls in a controlled environment and subsequently characterized their microstructure and mechanical properties. Following this, slot milling operations were conducted to explore the impact of spindle speed (*s*) and machining direction, as represented in Figure 18, where roughness profiles were measured in the centre of the grooves. It was noted that the WAAM process yielded a fine dendritic microstructure. Lava phases and carbides were also detected in the interdendritic regions and attributed to non-equilibrium cooling conditions [90]. At higher *s* values, lower *R*_a_ and torque (*M*) values were obtained. The best *R*_a_ = 0.258 μm was achieved within the studied cutting parameters when milling with *s* = 60 m/min in the building direction. The anisotropy of WAAMed INCONEL^®^ 718 alloy influences its machinability and residual stresses [91]. Milling along the extruder travel direction offers better dimensional tolerance values with lower cutting *M*, which is more efficient.

**Figure 19:**
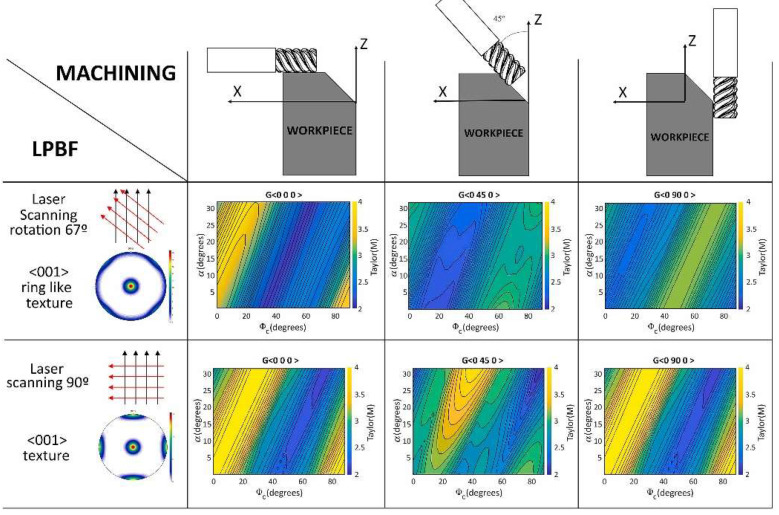
Interaction between laser-powder bed fusion (LPBF) and machining processes through orientation distribution function (ODF) patterns [92].

Pérez-Ruiz et al. [92] systematically investigated the impacts of LPBF parameters on *F*_c_ and the anisotropy of INCONEL^®^ 718, both theoretically and experimentally. A Taylor-based model for oblique-cutting was introduced to quantitatively assess the crystallographic effects. Peripheral milling operations were conducted across 54 experiments to analyze the relationships between machining parameters, layer thickness and the microstructural characteristics of 3D-printed alloys. The directional dependency of milling forces was linked to the interaction between the plane of the shear band and the orientation and size of the LPBF-printed columnar grains (Figure 19). The low-volumetric energy density (VED) manufacturing conditions increase the grain boundary density. Considering the possible development of the grain boundary sliding mode for small grains (<10 μm), *F*_c_ was lower when the tool axis was parallel to the columnar grain central axis.

Conversely, the highest *F*_c_ values occurred when the tool position generated planes of the shear bands transverse to the major axis of the columnar grains. Soffel et al. [93] examined the production and restoration of INCONEL^®^ 718 components using casting, interface milling and direct metal deposition (DMD). Four processing routes were explored, with variations in heat treatment and interface conditions, before implementing the DMD process. The cast components undergo either solution annealing or remain untreated, and the interface to the DMD part is maintained either in its as-cast state or subjected to milling. The sequentially processed INCONEL^®^ 718 specimens demonstrate seamless transitions from the cast section to the DMD material without fusion defects. The tensile properties of specimens extracted from the transition zone were *σ*_y_ = 409 MPa and *σ*_u_ = 782 MPa, exceeding the level of as-cast material: *σ*_y_ = 354 MPa and *σ*_u_ = 750 MPa, although *ε*_u_ is reduced from 32 to 24%. In 11 out of 12 instances, the fracture occurred within the cast section, indicating that the condition of the direct metal deposition (DMD) structures, whether milled or subjected to a sand-blasted interface, on both as-cast or solution-annealed INCONEL^®^ 718, is not a critical factor for part damage under static tensile load. The tensile properties of DMD material are moderately anisotropic; the horizontal specimens had *σ*_y_ = 561 MPa and *σ*_u_ = 909 MPa with *ε*_u_ = 31%, showing +7.9% *σ*_y_, −2.5% *σ*_u_ and +10.7% *ε*_u_ compared with the vertical test specimens that obtained *σ*_y_ = 520 MPa, *σ*_u_ = 932 MPa, *ε*_u_ = 28%, and were extracted parallel to the building direction (vertical specimens).

**Figure 20:**
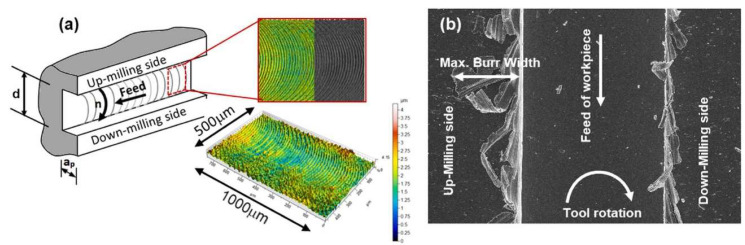
(**a**) Evaluation of surface roughness (SR) in micro slots. (**b**) Measurement of burr width at slot edges [95].

Arlyapov et al. [94] examined a metal–matrix composite (MMC) milling process based on INCONEL^®^ 625 with added NiTi-TiB2. Parameters such as *s*, feed per tooth (*f*_z_), axial depth (*a*_e_) and *a*_p_ are optimised, while *VB*_max_ and *R*_a_ were minimised. The MMC manufactured by DLD was efficiently machined with *s* = 20–30 m/min, *f*_z_ = 0.03–0.05 mm/tooth and the critical *VB* values varied from 110 to 115 μm. Subsequent to the *VB* region, there was a notable surge in the applied forces, followed by a brittle fracture of the tooth and heightened wear and deterioration of the cutting tool. Milling with *s* = 25 m/min speed ensured 28 min of stable operation. Afterwards, *VB =* 110 μm was reached. For *s* = 50 m/min, *VB* = 110 μm was reached and elapsed *Δt* = 14 min. Danish et al. [95] studied micro-milling on AMed INCONEL^®^ 718 under different sustainable cooling conditions. TW, *R*_a_ and burrs generated during micro-milling experiments were examined (Figure 20) under the influence of air, minimum quantity lubrication (MQL) and emulsion flood-cooling (EFC) conditions. The findings revealed that MQL conditions offer appropriate cooling and lubrication effects, leading to minimal TW and enhanced TL by nearly +45% compared to dry conditions. MQL also reduced *R*_a_ by approximately 65% less than in dry conditions. Other machining characteristics, including burr width and *F*_c_, also demonstrated improvements in both MQL and EFC environments. Scanning electron microscopy (SEM) micrographs and the EDX spectrum analyses revealed that the abrasive and adhesive wear were the governing wear mechanisms and that the lubricating environments did not affect the exact mechanisms of the cutting tools. However, TW was discovered to have a significant impact on burr development. Milling in a chilled air environment did not yield substantial enhancements in terms of machining characteristics.

**Figure 21:**
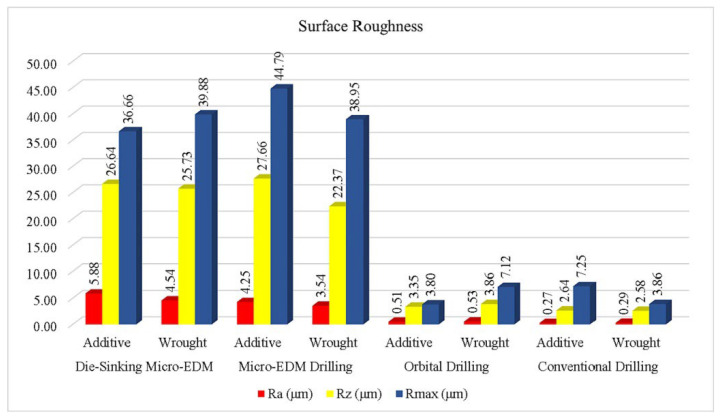
Comparison of surface roughness (SR) values for INCONEL^®^ 625 alloy specimens, whether wrought or produced through wire arc additive manufacturing (WAAM), drilled using die-sinking micro-electrical discharge machining (EDM), micro-EDM drilling, orbital and conventional drilling methods [96].

Ceritbinmez et al. [96] examined the drilling processes of wrought and WAAMed INCONEL^®^ 625 samples using die-sinking micro-EDM, conventional micro-EDM, orbital drilling and conventional drilling (Figure 21). Thermal drilling methods formed a white layer with *t* = 20–25 μm and *t* = 35–50 μm in the cross-sections of wrought and WAAM specimens, respectively. This phenomenon was not observed in the conventional drilling methods. *R*_a_ values inside the hole were most diminutive in the conventional drilling process, with a difference of 46.15%, 94.62% and 92.82% compared to the orbital, die-sinking and micro-EDM methods, respectively, due to drill cutting form, and since the helix angle used in this method facilitated chip evacuation. For wrought INCONEL^®^ 625 samples, conventional drilling registered *R*_a_ = 0.27 μm, orbital drilling *R*_a_ = 0.51 μm, die-sinking *R*_a_ = 4.54 μm and micro-EDM drilling *R*_a_ = 3.54 μm. For AMed INCONEL^®^ 625 samples, conventional drilling registered *R*_a_ = 0.29 μm, orbital drilling *R*_a_ = 0.53 μm, die-sinking *R*_a_ = 5.88 μm and micro-EDM drilling *R*_a_ = 4.25 μm. This study enhanced the application of a traditional process (drilling) to a non-conventional process (WAAM).

**Figure 22:**
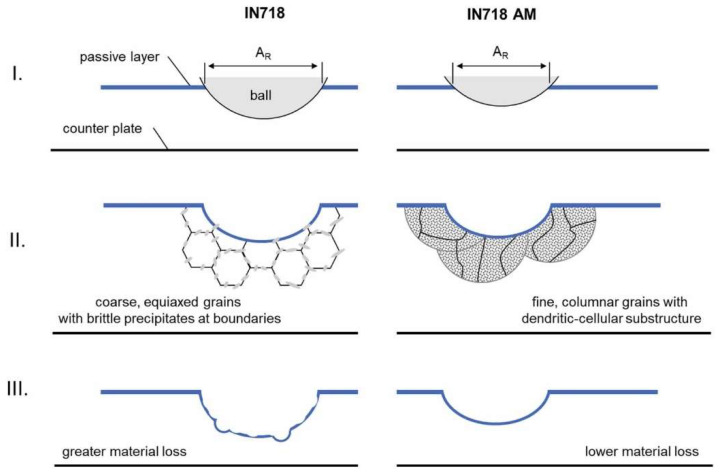
The tribocorrosion model is founded on the distinct structures of the examined materials, where stages I, II and III represent different phases in the material loss process [97].

Stachowiak et al. [97] assessed wear attributed to friction and tribocorrosion to identify the predominant wear mechanism under varying ball loads for LPBF and cast INCONEL^®^ 718. AMed INCONEL^®^ 718, fabricated through the LPBF method, exhibited superior tribocorrosion resistance in an environment with 3.5% NaCl and demonstrated enhanced mechanical wear resistance compared to cast INCONEL^®^ 718, owing to its microstructure. The uniform and finely columnar-grained microstructure with a dendritic-cellular substructure ensured a reduced surface area of the substrate material exposed to frictional interactions (Figure 22). The increase in load within the contact zone between the ball and the sample amplified tribocorrosion and purely mechanical wear. In cases of friction-only interactions, this relationship aligns with the Archard model [98].

#### Thermally Assisted Machining (TAM)

Induction-assisted machining (IAM), laser-assisted machining (LAM), plasma-assisted machining (PAM), laser belt processing (LBP) and flame-assisted machining (FAM) are hybrid machining processes that employ additional sources of energy to soften the workpiece, thus improving the efficiency and quality of the CM process. IAM uses eddy currents created by magnetising and coercive forces [99] to heat the workpiece locally, softening the material and reducing the machining *F*_c_ required [100]. LAM uses a concentrated laser beam that heats in a small vicinity to soften and consequently improve the workability and material removal earlier in its machining [101], which results in smoother and more precise cuts. PAM uses a plasma arc to generate heat and ionise the gas, which enhances the chemical reactions and MRR. LBP uses a laser beam to heat and melt a narrow strip of the workpiece, which is then rapidly cooled to induce compressive stresses that improve the surface’s wear resistance and fatigue life. FAM uses a fuel gas and an oxidising gas to generate a flame that heats and melts the material, which is then removed by a high-velocity gas stream [100].

Venkatesan [102] trialled the influence of LAM input variables on the machinability of INCONEL^®^ 718. The machining characteristics were compared to analyze the process advantages under varying laser machining parameters. The LAM input parameters are *v*_c_ = 60–150 m/min, *f* = 0.05–0.125 mm/rev and laser power (*P*_Laser_) between 1200 and 1300 W. Output results such as *F*_c_, *R*_a_, TL and geometrical characteristics of the chip are investigated and compared with conventional turning (CT) without application of laser heating. The DOE method is applied to the experiment using an *L*_16_ orthogonal array. *F*_c_, *R*_a_ and subsurface damage have significantly reduced under laser-assisted turning (LAT) compared to CT.

**Figure 23:**
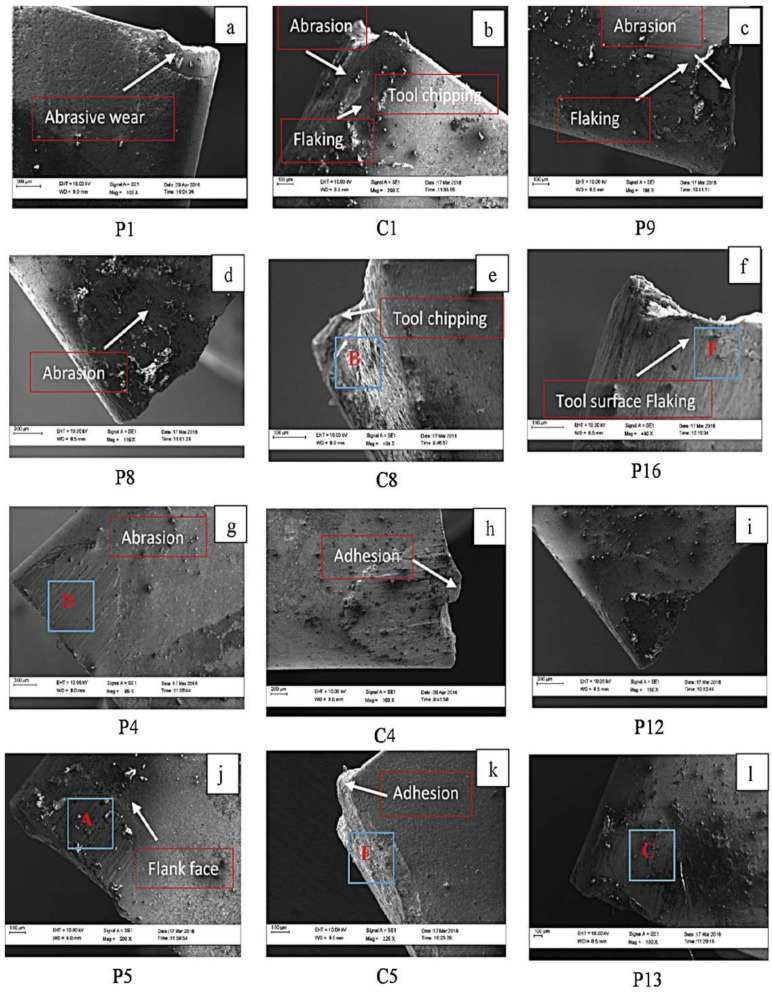
Scanning electron microscopy (SEM) images of *VB* were captured under different cutting conditions: P1, P9, P8, P16, P4, P12, P5, P13 for laser-assisted cutting, and C1, C8, C4, C5 for CT [102].

On the other hand, TL was severely improved. *P*_Laser_ = 1200 W is the optimum laser power at *f* = 0.125 mm/rev, *a*_p_ = 0.5 mm and *v*_c_ = 150 m/min with carbide tooling. Employing this *P*_Laser_ value, LAT provides 2.1× less *F*_c_, 46% reduction in *R*_a_ and 66% improvement in TL without subsurface damage, compared to CM (Figure 23) using *VB*_max_ = 300 μm [37] as a base value to recognise practical TL. During chip formation, LAT exhibited an increase in chip thickness (*h*), a decrease in shear angle and a reduction in saw-tooth pitch compared to CT. TW mechanism analysis in CT revealed abrasion, flaking, catering and edge chipping, whereas LAT showed only abrasion and flaking, suggesting that the LAT process alleviates the pronounced surface work hardening effect.

**Figure 24:**
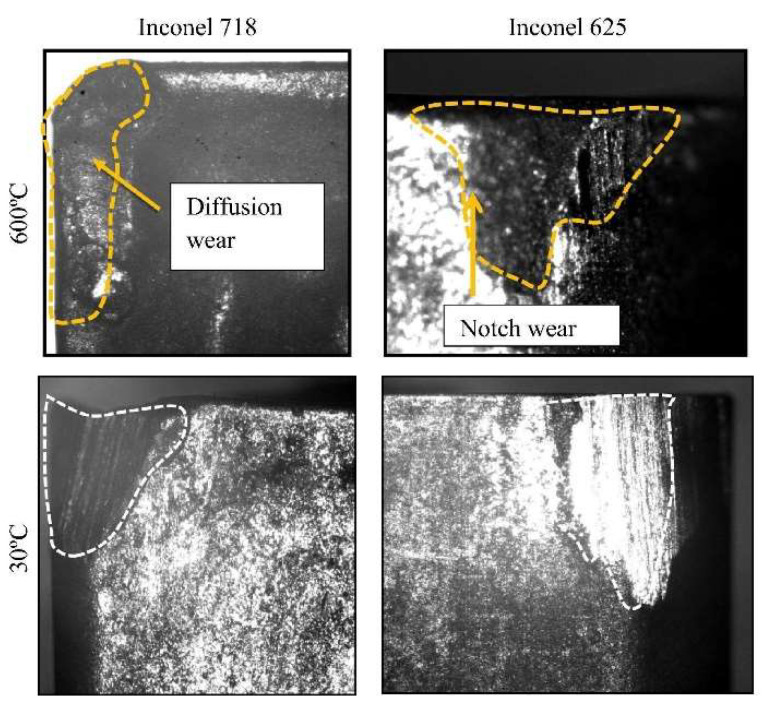
Tool wear (TW) on machining INCONEL^®^ 718 and 625 at *v*_c_ = 100 m/min, *f* = 0.13 mm/rev at room and heating *T* = 600 °C [103].

Parida and Maity [103] studied chip formation in the turning process of three Ni-based superalloys (INCONEL^®^ 718, INCONEL^®^ 625 and MONEL^®^ 400) using FAM. Parameters such as *F*_c_, TL, chip morphology, TW and surface integrity (including *R*_a_ and microhardness beneath the machined surface) were investigated at room and elevated temperatures (300 °C and 600 °C). Only the INCONEL^®^ 718 and 625 alloys will be addressed in this state-of-the-art review. Focusing on the two first alloys, the TL of machining of INCONEL^®^ 718 was lower compared to INCONEL^®^ 625 due to *k*; however, the surface finish of INCONEL^®^ 718 was the best. The highest *F*_c_ was obtained for INCONEL^®^ 718 and cutting feed force (*F*_x_) for INCONEL^®^ 625. The chip formed with the heating condition for INCONEL^®^ 718 was coiled and spiral, whereas, for INCONEL^®^ 625, it was coiled and straight. INCONEL^®^ 718 experiences the highest cutting zone *T*. For a heating *T* = 600 °C, when machining INCONEL^®^ 625, the tool suffered from notch wear, whereas for INCONEL^®^ 718, crater and diffusion wear were noted (Figure 24). The microhardness beneath the FAMed surfaces of INCONEL^®^ 718 and 625 is lower than that of CT due to heat application.

**Figure 25:**
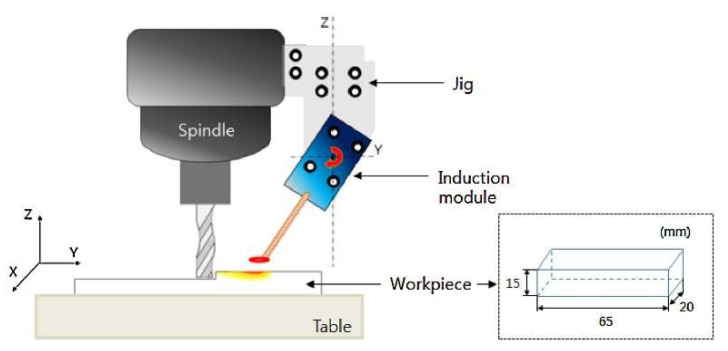
Schematic of laser induction-assisted machining (LIAM) applied to the workpiece with a flat shape [104].

Baek et al. [104] analysed and experimentally investigated two other approaches, IAM and laser induction-assisted machining (LIAM), as schematised in Figure 25, to compare their efficiency with CM for AISI 1045 steel and INCONEL^®^ 718. In this state-of-the-art review, only the INCONEL^®^ alloy will be addressed. During the milling process, when *s* increased, *F*_c_ was reduced at all depths during machining. *F*_c_ was decreased by up to 70% when milling INCONEL^®^ 718 with IAM, compared to CM. Moreover, the same *F*_c_ decreased by up to 67% when milling INCONEL^®^ 718 with LIAM, compared with IAM. LIAM offers the lowest values of *F*_c_. When milling INCONEL^®^ 718 in CM, *VB* = 288 μm and *R*_a_ = 0.711 μm; in IAM, *VB* = 341 μm and *R*_a_ = 0.437 μm; and LIAM *VB* = 225 μm and *R*_a_ = 0.281 μm, demonstrating LIAM as an excellent hybrid manufacturing process to facilitate INCONEL^®^ 718 machining.

**Figure 26:**
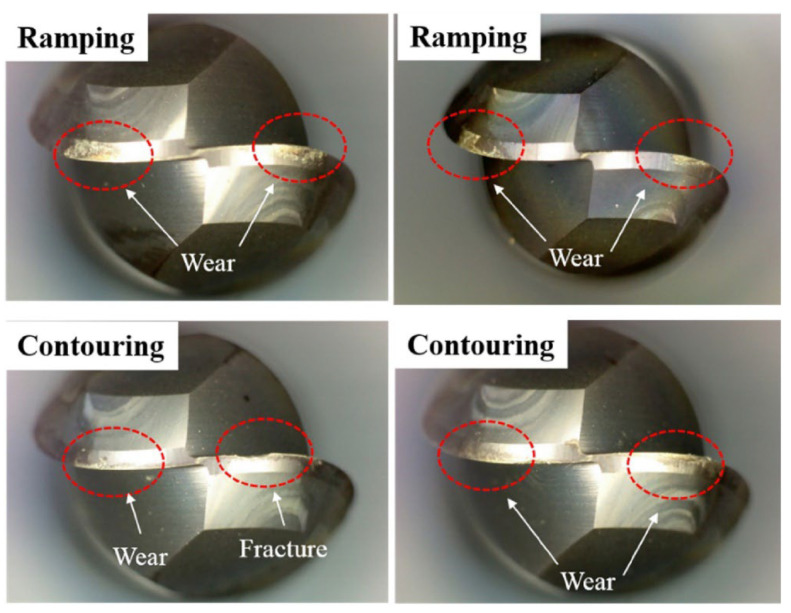
Tool wear (TW) in INCONEL^®^ 718 conventional manufacturing (CM) and induction-assisted machining (IAM) [105].

Choi and Lee [105] performed IAM on circular cone shapes made of AISI 1045 steel and INCONEL^®^ 718. Ramping and contouring milling methods were experimented with both materials to assess *F*_c_, *R*_a_ and TW (Figure 26). Finite element analysis (FEA) determined the effective depth of cut (EDOC or effective *a*_p_). In this state-of-the-art review, only the INCONEL^®^ alloy will be addressed. For INCONEL^®^ 718, effective *a*_p_ = 0.3 mm below the surface at the selected optimum preheating *T* = 900 °C. The maximum efficiency of IAM was reached in this experiment when *f* = 50 mm/min, and it was confirmed that *f* is an essential parameter in IAM to improve machining characteristics. Compared to CM, when milling INCONEL^®^ 718 with IAM, *F*_c_ and *R*_a_ were decreased by 8.73–34.3% and 16.3–45.2%, respectively, thanks to the softened material and decreasing tool vibration. Compared to CM, in ramping and contouring INCONEL^®^ 718 with IAM, *F*_c_ decreased by 34.3% and 29.2%, respectively. IAM was demonstrated to be superior in efficiency for machining difficult-to-cut materials.

Moon and Lee [106] developed and investigated a PAM process in this study, comparing its performance with LAM and CM. The proper preheating *T* and *a*_p_ were determined by thermal analysis. Experiments were conducted using the determined preheating *T* and *a*_p_ with the PAM system on AISI 1045 steel and INCONEL^®^ 718. In this state-of-the-art review, only the INCONEL^®^ alloy is addressed. The thermal analysis established the effective *a*_p_ for the PAM and LAM machining conditions. When the angle of the plasma torch was 70°, the proper preheating effect was obtained. For INCONEL^®^ 718, with PAM, *F*_c_ and *R*_a_ decreased by 57 and 82%, respectively, compared with CM, although *F*_c_ and *R*_a_ increased by 9 and 4%, respectively, compared with LAM. The results showed that PAM and LAM are excellent choices for hybrid manufacturing; however, since the plasma torch is cheaper than the laser beam machine, PAM is considered a more efficient method, considering manufacturing costs.

**Figure 27:**
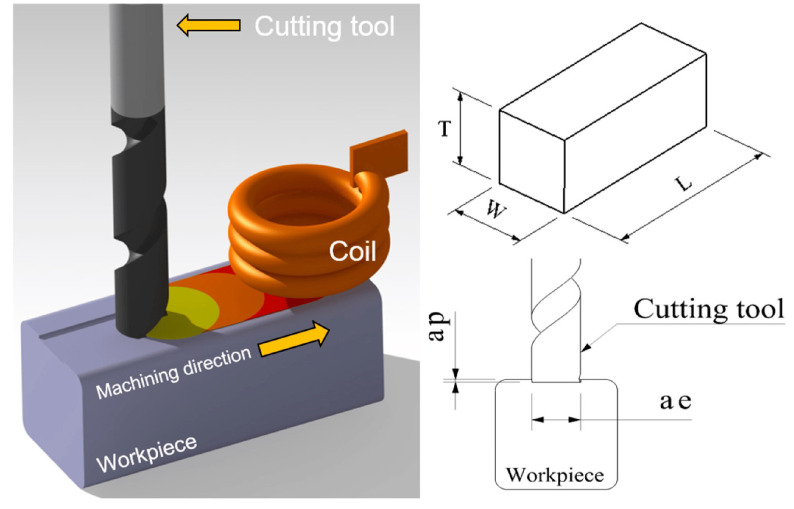
The schematic diagram of induction-assisted machining (IAM) [107].

Kim and Lee [107] examined the IAM preheating technique for INCONEL^®^ 718 utilizing a magnetic induction coil (Figure 27) and assessed its impact on both coated [108,109] and uncoated cutting tools. Prior to machining experiments, FEA was conducted to determine the optimal *a*_p_. The study focused on critical parameters, including *F*_c_, *R*_a_ and TW, optimizing machining conditions based on stress/number of cycles (S/N) ratio, ANOVA and response optimization techniques. FEA results indicated the maximum *a*_p_ = 0.25 mm when the preheating temperature was set at 950 °C. Optimal parameters obtained through DOE revealed that for the coated tool, using *s* = 8000 rpm, *a*_p_ = 0.2 mm and *f =* 150 mm/min n achieved *R*_a_ = 0.205 μm. Meanwhile, the uncoated tool, at *f* = 100 mm/min, did not register an *R*_a_ value. Ultimately, the coated tool demonstrated superior efficiency compared to the uncoated tool. Vignesh and Ramanujam [101] evaluated the impact of laser-assisted high-speed machining (LAHSM) on the *F*_c_, *R*_a_, TW and chip morphology in the turning process of INCONEL^®^ 718. *P*_Laser_ = 1300 W, *v*_c_ = 80 m/min and *f* = 0.08 mm/rev were set as optimal parameters, having obtained *R*_a_ = 0.328 μm. Thus, the *F*_c_, *R*_a_ and TW values were better over CT, reducing *F*_c_, *R*_a_ and TW by 24.5%, 56% and 29%, respectively.

**Figure 28:**
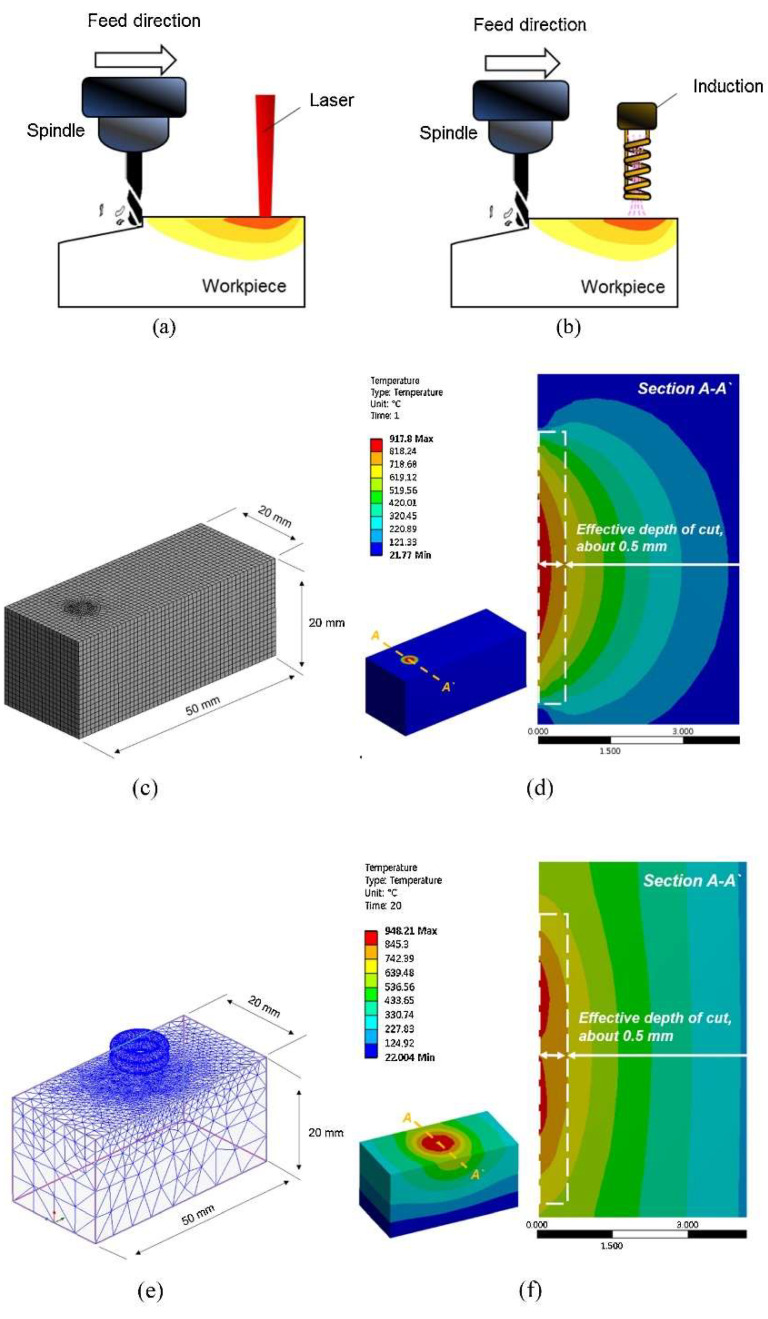
Schematic diagram of (**a**) laser-assisted machining (LAM), (**b**) induction-assisted machining (IAM), (**c**) finite element analysis (FEA) model and (**d**) results of laser thermal induction; (**e**) FEA model and (**f**) results of magnetic induction (adapted from [110]).

Kim and Lee [110] assessed machining efficiency by power consumption (*P*_in_) in INCONEL^®^ 718 machining. CM, LAM and IAM were considered (Figure 28a,b). Numerical thermal and thermal-electromagnetic analyses were performed via ANSYS^®^ software (https://www.ansys.com/) to verify the effective *a*_p_ and dwell time for preheating (Figure 28c–f). *P*_in_, *F*_c_ and *R*_a_ were analysed according to the various machining conditions. FEA was performed to determine dwell time and effective *a*_p_. The analysis showed that the optimum dwell time was 20 s and effective *a*_p_ = 0.5 mm. *P*_in_ increased as *f* increased in LAM and IAM, primarily due to the need to maintain the preheating *T*. Namely, IAM registered the highest *P*_in_ since it needed a longer preheating time thanks to the lower thermal energy concentration. Comparing CM to LAM, there was a 32% *P*_in_ increase and an *F*_c_ and *R*_a_ decrease of 41 and 51%, respectively. Comparing CM to IAM, there was a 66% *P*_in_ increase and an *F*_c_ and *R*_a_ decrease of 45 and 32%, respectively. It was noted that LAM and IAM consume more power than CM; nonetheless, machinability was improved, and LAM was considered the most suitable hybrid process within the scope of this paper.

**Figure 29:**
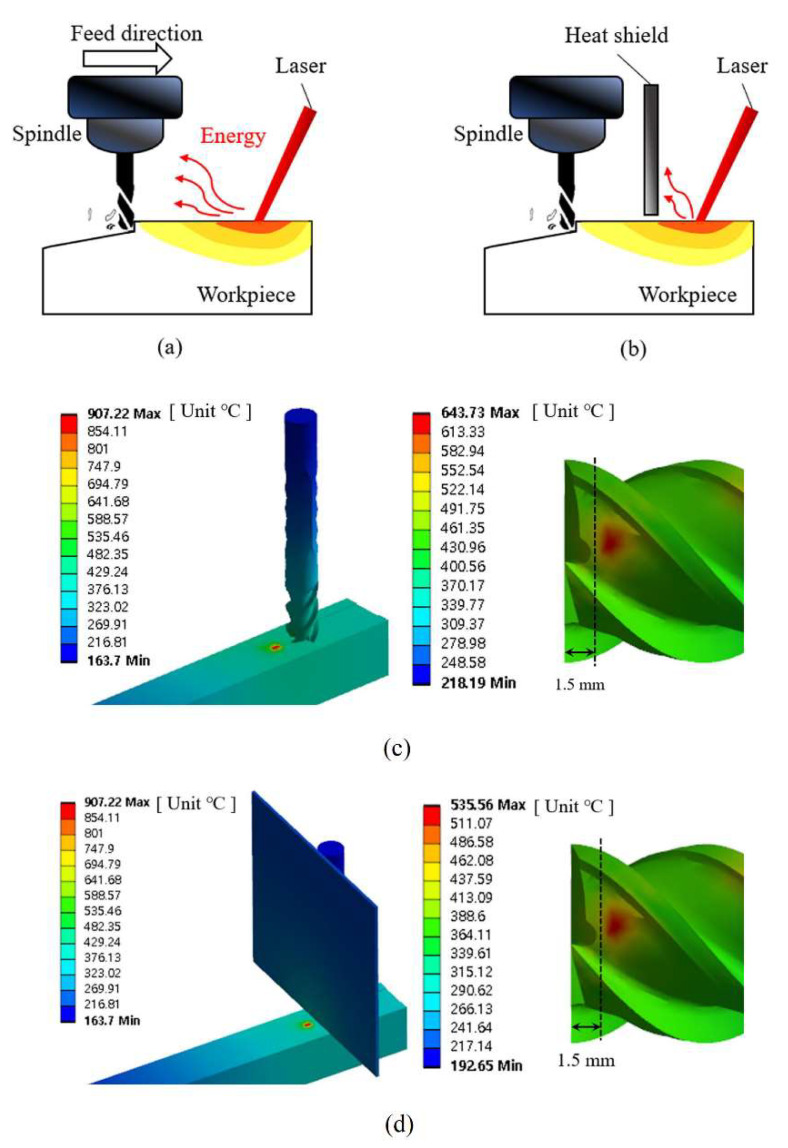
Conceptual diagram of (**a**) laser-assisted machining (LAM) and (**b**) LAM with heat shield, (**c**) thermal finite element analysis (FEA) on LAM, (**d**) thermal FEA on LAM with heat shield (adapted from [111]).

Jeong and Lee [111] dramatically improved INCONEL^®^ 718 machinability using LAM with a heat shield (Figure 29a,b). TL experiment analysis was carried out under the same MRR according to the application of the heat shield. A numerical thermal analysis was performed to determine the adequate *a*_p_. TL changes and machining efficiency due to heat shield application and effectiveness are detailed. It was found that INCONEL^®^ 718 processing quality was higher when the heat shield was applied with LAM. The thermal analysis determined that *a*_p_ = 0.3 mm with a preheating *T* = 900 °C. *T* of the tool, without the heat shield, was 643.73 °C (Figure 29c), whereas, with the shield, it was 535.56 °C (Figure 29d), reducing the escaped heat from the laser to the tool.

Regarding TL, LAM increased it by about 53%, compared to CM. On the other hand, LAM + shield increased TL by about 78.3% compared to CM. Thanks to the heat shield, fewer abrasions, cracks and fractures were measured because the tool was protected from parasitic heat that would soften it. The authors concluded that LAM with a heat shield can be used to obtain a better product and TL.

**Figure 30:**
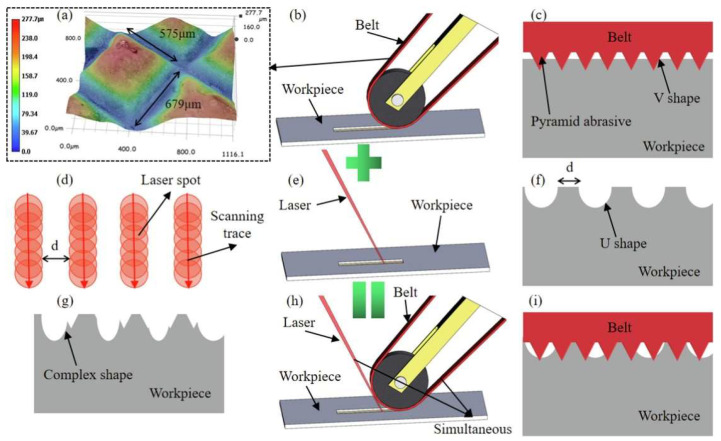
Principle of laser belt processing (LBP) and preparation of a grooved surface: (**a**) pyramid abrasive; (**b**) belt grinding; (**c**) micro-groove structure ground by belt; (**d**) laser scanning trajectory; (**e**) laser processing; (**f**) microgroove structure processed by laser; (**g**) microgroove structure processed by laser belt; (**h**) laser belt processing; (**i**) laser belt processing process [112].

Liu et al. [112] proposed a new and efficient method, LBP (Figure 30), to prepare INCONEL^®^ 718 surfaces with ultrahigh adhesion and anisotropic wettability. Microgroove depth by LBP effectively increased with a higher *P*_Laser_. When *P*_Laser_ reached considerable values, errors were observed in the width and spacing of the microgrooves due to excessive ablation at high *P*_Laser_. *R*_a_ values increased for higher *P*_Laser_. The *R*_a_ values slightly reduced when *P*_Laser_ = 9.6 W, caused by molten material movement before resolidification. Greater MRR occurred with extended processing times, increasing microgroove depth and *R*_a_. However, when processing times were further increased to 8×, microgroove depth and *R*_a_ diminished due to additional grinding by the pyramid abrasive grains on the belt. In comparison to conventional belt grinding, LBP offers superior surface quality.

**Figure 31:**
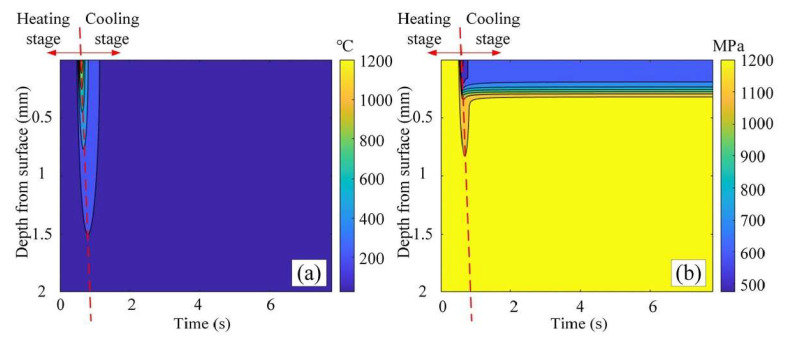
Distribution of *T* and *σ*_y_ along the depth direction during laser scanning [113].

Zhang et al. [113] applied LAM to enhance the machinability of INCONEL^®^ 718, focusing on the heat-affected zone (HAZ) as the core of the research. This study further elucidates the yield strength distribution within the HAZ, presenting a theoretical model for the yield strength gradient during the LAM of INCONEL^®^ 718. The proposed model is developed by considering the workpiece’s strengthening mechanisms, microstructure and *T* distribution. It anticipates the HAZ range and provides a quantitative depiction of the spatial gradient of *σ*_y_ in the HAZ (Figure 31a, illustrating the decrease in T amplitude at different depths as the distance from the surface increases). Compared with the Johnson–Cook (JC) equation, the proposed model is more practical and effective in predicting the HAZ *σ*_y_ and determining the range of the HAZ *σ*_y_ in LAM (Figure 31b, as time changes, *σ*_y_ at different depths decreased first, and then increased slightly with the temperature change). Since the error between the *σ*_y_ at room *T* obtained by the proposed model and the experimental data is within 5% tolerance, the predicted results agree with the experimental results. It was also found that the *σ*_y_ gradient decreases with the increasing *P*_Laser_ and decreasing laser scanning speed. In the heating process, when *T* < 700 °C, the contribution of the precipitation strengthening component (*σ*_p_) to *σ*_y_ exceeds 50%, followed by the solid solution strengthening component (*σ*_SS_), grain boundary strengthening component (*σ*_D_) and Ni-matrix strength component (*σ*_Ni_). With the increasing *T* amplitude, *σ*_p_ decreased significantly, *σ*_SS_ increased and *σ*_D_ and *σ*_Ni_ continued to decrease slowly. *σ*_D_ and *σ*_Ni_ cause the slow increase in *σ*_y_ at the cooling stage.

#### Ultrasonic Machining (USM)

**Figure 32:**
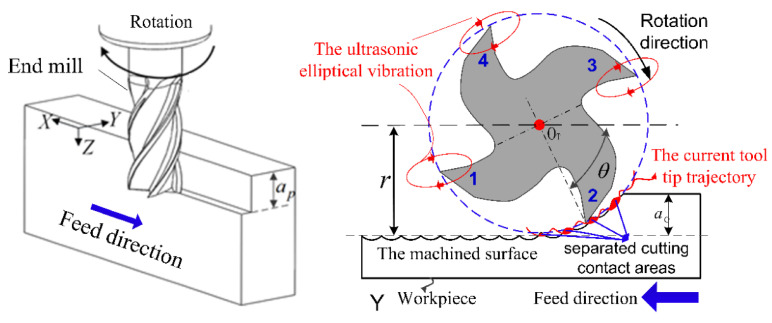
Ultrasonic peening milling (UPM) process illustrated in a four-flute end milling cutter scheme (adapted from [117]).

USM [100,114] can be divided into various variants. Ultrasonic-assisted turning (UAT) is a variant of USM that combines the benefits of USM with the versatility of a lathe. It involves using high-frequency vibration, above 16 kHz, inflicted on the cutting tool during machining. It is known for producing high-quality surfaces with minimal TW. The high resonance frequency and low amplitude are used for higher MRR [115]. High-speed ultrasonic vibration cutting (HUVC) is a modification of USM that uses a high-speed spindle to increase the machining speed and provides TL extension by up to 6×, compared to CM approaches [116]. Hot ultrasonically assisted turning (HUAT) is a technique that uses a heated workpiece (with LAM, for example) and ultrasonic vibration to improve the machinability of difficult-to-machine materials. It significantly improves machinability by reducing *F*_c_ and effective stress, but *T* increases compared to CM and UAT [30]. Ultrasonic peening milling (UPM, Figure 32) is a process that uses vertical ultrasonic vibration to enhance the surface properties of a workpiece. Ultrasonic vibration-assisted milling (UVAM) is a technique that combines the benefits of USM with the efficiency of milling [117], as shown in Figure 32.

Suárez et al. [42] compared the effects of alternative manufacturing processes, such as AWJM, WEDM and UVAM, with CM (Figure 10). Surface integrity, hardness, residual stress and fatigue strength obtained from these machining processes have been examined for cutting INCONEL^®^ 718. Regarding UVAM, the input parameters are *s* = 2123 rpm, *f* = 254 mm/min, *a*_e_ = 6 mm, *a*_p_ = 0.17 mm, vibration amplitude (*VA*) of 1.51 μm and a vibration frequency (*VF*) of 39.6 kHz. The tool used has a diameter (*Ø*_T_) of 12 mm and four teeth. UVAM produced the smoothest surface samples with *R*_a_ = 0.2 μm, although the highest surface hardness was observed compared to pure non-conventional processes and CM (2 HRC higher than the last one referred). UVAM surfaces demonstrate CRS profiles. The UVAM sample surface had the highest fatigue strength from the four-point bending test. UVAM shows the best results compared to other manufacturing processes addressed within the paper regarding the *R*_a_ and fatigue strength tests.

Peng et al. [118] studied the influence of HUVC on the machining of INCONEL^®^ 718 by combining cutting operations with mechanical surface treatment in a single step. The conducted experiment aims to contrast the surface integrity achieved through CM and HUVC utilizing coated carbide tools at *v*_c_ = 80–240 m/min. Compared to CM, HUVC improved *R*_a_ by 22.45%, producing a regular surface micro-texture, deeper subsurface deformation zone between 50.21 and 104.47 μm (CM reaches between 28.02–61.92 μm), and a higher surface CRS from −1772 to −2323 MPa (compared to CM from −912 to −1434 MPa). It was also observable that, as *v*_c_ increased, the surface micro-hardness along the *f* direction gradually increased in CM and HUVC. Occasionally, a singular HUVC step may suffice to meet the initially addressed requirements encompassing both cutting operations and mechanical surface treatment, thereby enhancing surface integrity. Zhang et al. [116] researched the effect of HUVC on INCONEL^®^ 718 turning, utilizing a tool equipped with a WC insert and employing the TGRA method to assess various outcomes. *v*_c_ = 80–300 m/min was identified as the stable cutting range for INCONEL^®^ 718. Furthermore, the cutting efficiency showed notable improvement with HUVC compared to the CT process.

**Figure 33:**
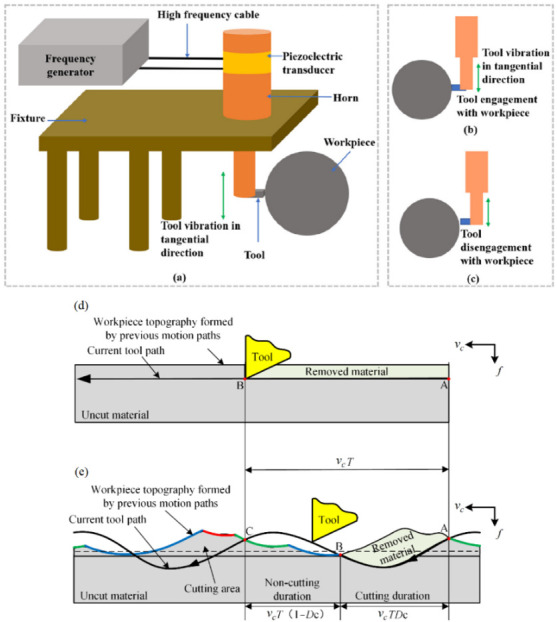
(**a**) Schematic representation of the ultrasonic-assisted turning (UAT) setup, (**b**,**c**) depiction of tool engagement and disengagement during the machining process, (**d**) a comprehensive comparison between conventional cutting and (**e**) high-speed ultrasonic vibration cutting (HUVC) within a single vibration cycle (adapted from [115,116]; caption: *T*—ultrasonic vibration period, *D*_c_—duty cycle).

Airao et al. [115] introduced a novel solution utilizing the UAT method (Figure 33) for INCONEL^®^ 718, incorporating various lubrication strategies, including dry, wet, MQL and CO2 (*l*). In the case of UAT with CO2 (*l*) as the cooling environment, it was established that *P*_in_ was reduced by 11 to 40%, obtaining an average *R*_a_ ≈ 0.95 μm. In contrast, the traditional turning process with the same cooling environment *P*_in_ was decreased only by 5 to 31%, achieving an average *R*_a_ ≈ 1 μm. Nonetheless, in UAT, *VB* was reduced by 32 to 53%, while in traditional turning, *VB* can be reduced by 32 to 60%.

Yin et al. [119] assessed TiAlN-coated carbide tools used in the experiment and compared TL, TW types and mechanisms in the different wear states in high-speed UPM and CM of INCONEL^®^ 718. The influence of different TW states on *R*_a_ and fatigue performance was systematically analysed. UPM applied to INCONEL^®^ 718 effectively reduces the oxidation wear of the tool in the initial wear stage and reduces both diffusion and adhered wear in the normal to severe wear stages.

**Figure 34:**
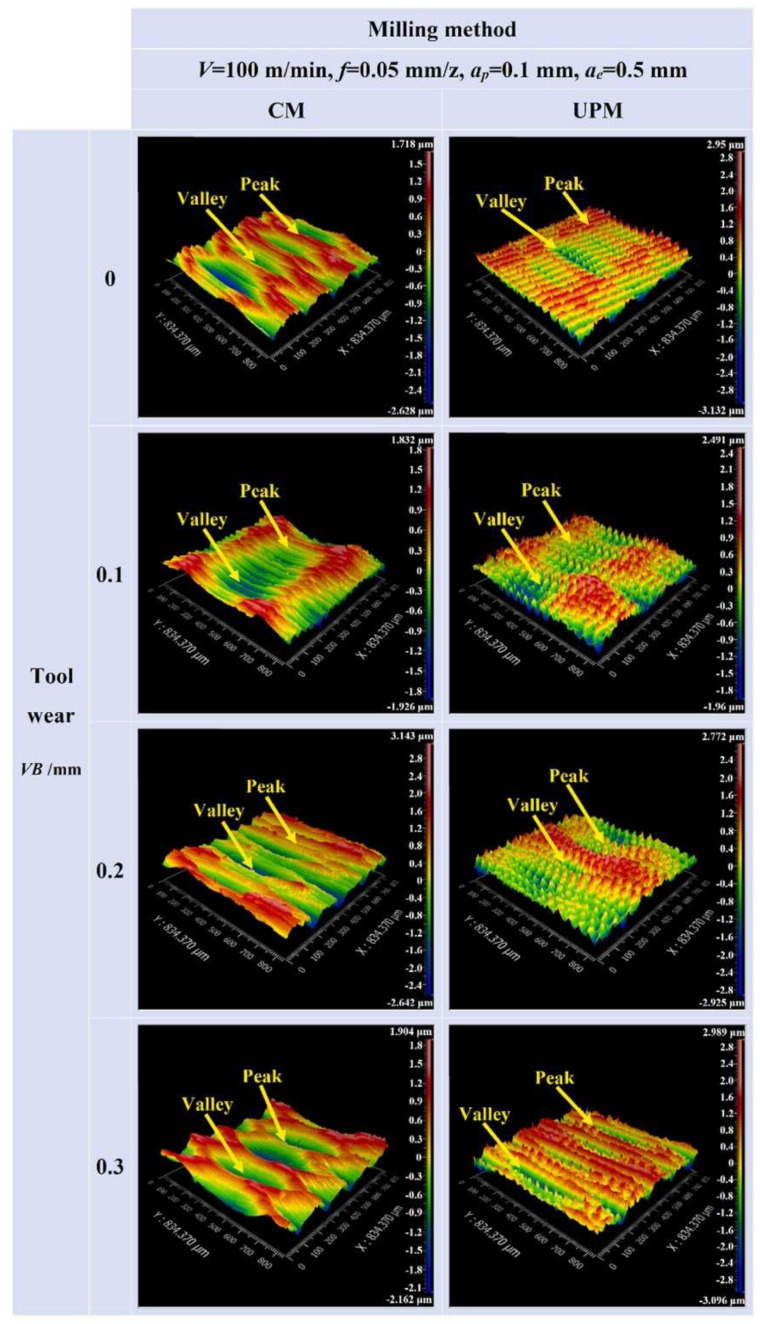
Comparison of surface topographies achieved through conventional manufacturing (CM) and ultrasonic peening milling (UPM) [119].

Furthermore, TL is extended by 32.5% compared to CM for *s* = 100 m/min. The surface quality is also improved, having observed a more uniform surface texture without surface defects in UPM (Figure 34). Nonetheless, it yields an elevated surface micro-hardness, increased surface CRS and a greater thickness of the micro-hardening layer, measured approximately 9.9–15.2 μm via UPM, whereas it resulted in 5.6–8.2 μm via CM. The hybrid process can considerably improve the fatigue life of INCONEL^®^ 718 since a workpiece made by UPM has a 17.12× higher fatigue life than CM under the same processing conditions.

**Figure 35:**
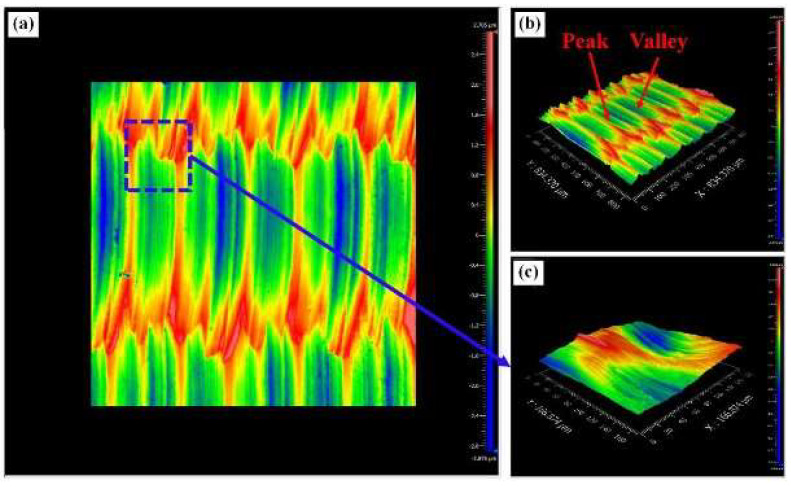
CMed 3D surface topography of S1: (**a**) surface measurement topography, (**b**) stereoscopic topography and (**c**) detailed topography; *v*_c_ = 40 m/min, *s* = 2124 rpm [120].

**Figure 36:**
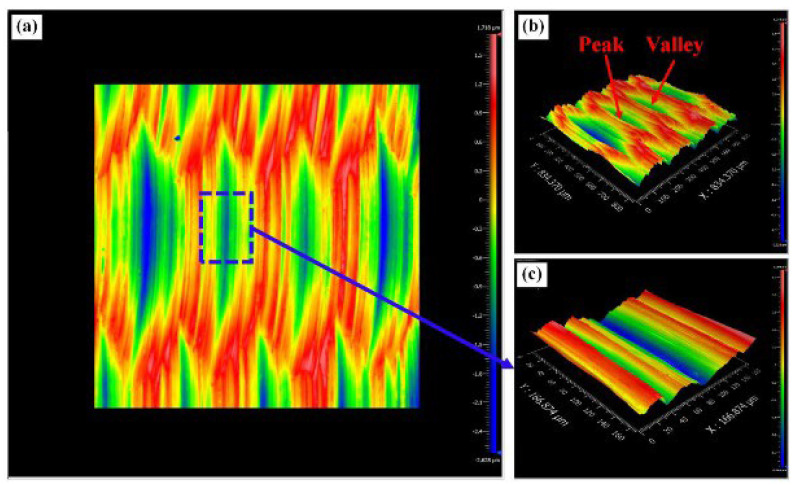
CMed 3D surface topography of S2: (**a**) surface measurement topography, (**b**) stereoscopic topography, and (**c**) detailed topography; *v*_c_ = 100 m/min, *s* = 5358 rpm [120].

**Figure 37:**
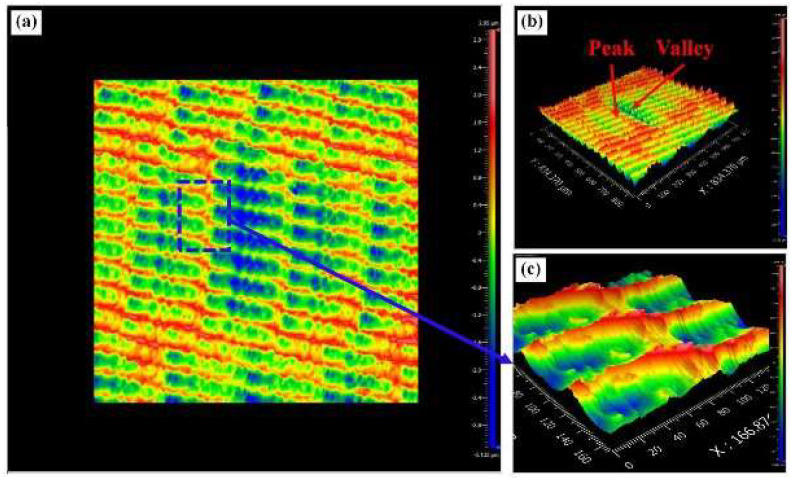
UPMed 3D surface topography of S3: (**a**) surface measurement topography, (**b**) stereoscopic topography, and (**c**) detailed topography; *v*_c_ = 100 m/min, *s* = 5358 rpm [120].

Yin et al. [120] studied UPM technology based upon INCONEL^®^ 718 machining to investigate the vibration’s effect on this material’s surface integrity and fatigue life, afterwards comparing the results with those obtained by CM. Compared with the CM (Figure 35 and Figure 36) processing of the INCONEL^®^ 718, UPM significantly altered the microstructure and roughness of the machined surface (Figure 37). With the same machining parameters, *R*_a_ = 0.4338 μm was obtained via CM; although *R*_a_ = 0.5928 μm was obtained via UPM, and it increased compared to CM, a uniform wavelike morphology with gentle peaks and valleys was visualised. UPM exhibited superior fatigue life, reaching 2.20 × 10^6^ cycles, 16.1× higher than CM, attributed to enhanced surface quality and reduced surface defects. Additionally, UPM increased the surface micro-hardness rate from 52.08% to 73.51%. The depth of the hardness layer in the cross-section increased from 65 to 80 μm, while the CRS changed from −229 to −819 MPa. Moreover, UPM resulted in a more extensive plastic deformation region with a *t* ≤ 15.2 μm.

#### 3.1.4. Laser Beam Machining and Laser Drilling Machining (LBM and LDM)

Laser beam machining (LBM) and laser drilling machining (LDM) are non-traditional and non-contact machining techniques that have gained significant attention recently. These techniques use a large high-energy plasma, or laser beam, in a small workpiece area to melt and vaporise the material from a workpiece that a gas jet or a vacuum removes, producing great MRR values [121]. It has several potential benefits over CM techniques: it can machine complex and intricate designs with high precision and accuracy; it can machine a wide range of materials, including metals, ceramics, plastics, and composites, with high-quality surface finish with minimal burrs and surface defects [122]; and it can be used to machine small and delicate parts that are difficult to machine using traditional techniques. Counterwise, thermal stresses, residual stresses and surface quality are the main disadvantages of this process [123]. LBM is applied in aerospace, automotive, medical and electronics applications. In the aerospace industry, LBM is used to machine complex and intricate designs for aircraft parts, such as INCONEL^®^ 718 and 625 turbine blades and engine components. The automotive industry uses LBM to machine components for engines, transmissions and fuel systems. LDM is a non-contact drilling process variant of LBM [71]. With the same operation proceedings, it can drill precise and accurate holes with high speed and efficiency; it can drill holes in a wide range of materials, including metals, ceramics, plastics, and composites; and it can drill holes with high aspect ratios, higher tapers [124] and complex geometries; and it can drill holes with minimal thermal damage and surface defects. It can also achieve higher MRR than electro erosion on drill holes for manufacturing applications. Like LBM, LDM is also applied in the aerospace and automotive industries.

**Figure 38:**
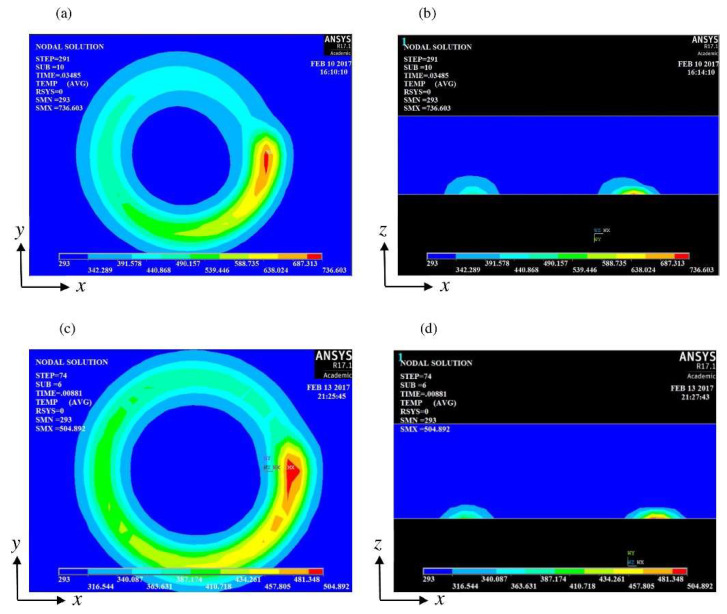
Top section view (**a**) and cross-section view (**b**) of the *T* field induced by a laser rotating at 3500 rpm. Top section view (**c**) and cross-section view (**d**) at a rotational speed of 7000 rpm, featuring a 0.2 mm radius and x-directional moving speed of 1000 mm/min (trochoidal path, *T* is expressed in the unit of Kelvin) [125].

Ahmed et al. [122] applied LBM on INCONEL^®^ 718 to study the optimal configuration of laser parameters and assess their impact on MRR and *R*_a_. The suggested optimal parameters include a pulse frequency (*f*_laser_) of 10 kHz, scan speed (*v*) of 341.41 mm/s and laser intensity (*I*) of 75%. These parameters were verified to yield an actual MRR (MRR_act_) approaching the designed value (MRR_th_) and *R*_a_ = 2.67 μm. Alsoruji et al. [124] proposed an enhanced machining procedure conducted in LDM using TGRA to determine optimal process factors for achieving improved MMR and *R*_a_ values. The optimum parameters for LDM included *P*_laser_ = 2 kW, *W*_D_ = 0.7 mm, a focal length of +2 mm and gas pressure (*P*_gas_) of 3 bar. Pan et al. [125] studied experimentally and characterised the melting zone shape in the LBM process of INCONEL^®^ 718 and the effects of laser scanning speed with a rotational path and *P*_Laser_ on the absorption ratio. A 3D FEA was proposed for the *T* field distribution prediction parallel to the experiment via ANSYS^®^ software (Figure 38). INCONEL^®^ 718 LBM predicted the coaxial laser preheating system *T* distribution field from FEA, which can represent a close approximation to the experiment. Melting zone area, depth and width (MZA, MZD and MZW, respectively) monotonically decrease with the increasing laser scanning speed for every *P*_Laser_ value used (400, 600 and 1000 W). Also, the increased *P*_Laser_ values could help to increase the melting zone area.

#### 3.1.5. Water-Jet Machining (WJM)

WJM is a non-traditional technique that uses a high-pressure water jet to cut, machine and shape materials [123]. The process is ideal for cutting ductile and sensitive materials, producing high-quality surfaces with minimal TW and material distortion. AWJM is a more advanced form of WJM that uses water and abrasive particles, usually made of garnet, Al_2_O_3_ or SiC, to machine hard-to-cut materials like Ti, steel and INCONEL^®^ alloys. WJM and AWJM have several advantages over CM techniques since they are non-thermal processes, thus implying the absence of thermal phenomena on the material’s surface. Thus, superficial micro-hardening is mitigated. The same processes are environmentally friendly, as they do not produce hazardous waste or emissions, offering high precision and accuracy, producing parts with tolerances as low as 10 μm [126].

**Figure 39:**
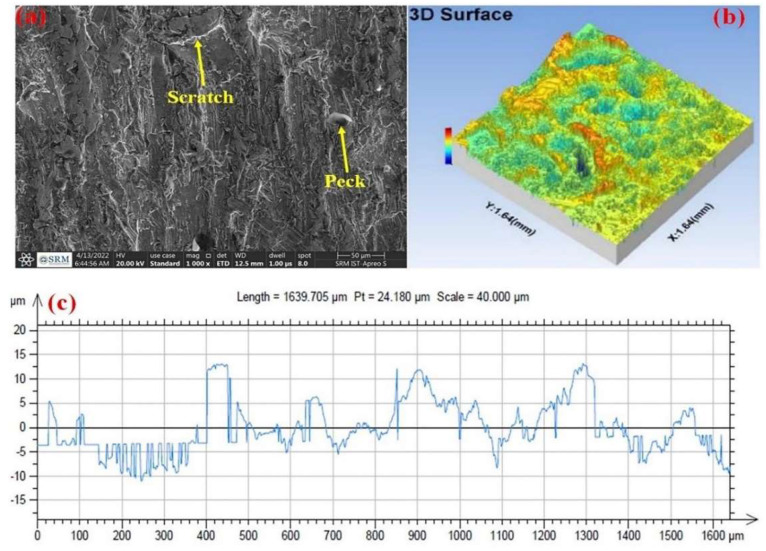
Desirability condition *P*_water_ = 300 MPa, *G*_d_ = 1 mm, *T*_SP_ = 72 mm/min, and abrasive material is constituted by 100% SiC. (**a**) Scanning electron microscopy (SEM) image, (**b**) 3D surface image, (**c**) 2D roughness profile image [130].

Suárez et al. [42] compared the effects of alternative manufacturing processes, such as AWJM, WEDM and UVAM, with CM (Figure 10). Surface integrity, hardness, residual stress and fatigue strength obtained from these machining processes have been examined for cutting INCONEL^®^ 718. Regarding WJM, the input parameters are *Ø*_N_ = 0.35 mm, water pressure (*P*_water_) of 350 MPa, *f* = 20 mm/min and abrasive material flow rate (*AM*_FR_) of 350 g/min. AWJM produced an intermediate-poor value of *R*_a_ = 1.6 μm from the four manufacturing processes studied. The surface hardness test also produced one of the lowest values. AWJM sample has a relatively shallow CRS state in the surface region. The relatively low penetration depth of 50 μm for AWJM could be the reason for the relatively low fatigue limit. Moreover, below this depth, the sample has a stress-free state. UVAM shows intermediate results compared to the other manufacturing processes addressed within the paper; however, it produces pieces with low surface hardness. Venkateshwar Reddy et al. [127] investigated the impact of standoff distance, *AM*_FR_ and traverse speed (*T*_SP_) on MRR, *R*_a_ and kerf width (*K*_W_) in the multi-response optimisation on the AWJM of INCONEL^®^ 625, employing ANOVA analysis. The study utilized weighted aggregates sum-product assessment (WASPAS) and multi-objective optimization based on ratio analysis (MOORA) methods. MRR exhibited a proportional relationship with *T*_SP_ and *AM*_FR_. *R*_a_ was influenced by *T*_SP_ and *AM*, improving with an increase in the former and decreasing with an increase in *T*_SP_. *K*_W_ was affected by all three input parameters, with standoff distance and *T*_SP_ having a particularly significant impact. SEM tests confirmed that higher *AM*_FR_ values resulted in higher *R*_a_, while lower *R*_a_ values were achieved with lower *AM*_FR_ under the same *T*_SP_ conditions. The optimum *R*_a_ = 4.7 μm was identified for *AM*_FR_ = 200 g/min and *T*_SP_ = 145 mm/min. Salinas et al. [128] addressed the influence of AWJM parameters on *R*_a_, topography, *a*_p_ and residual stress when milling INCONEL^®^ 718. The 3D optical microscopy and SEM techniques were used to analyse surface characterisation. The X-ray diffraction (XRD) technique was used to measure the residual stress field in the longitudinal and transverse directions. A concise and summarised table about the effect of AWJM parameters of INCONEL^®^ 718 was presented. *R*_a_ = 12.2–24.6 μm increased as *P*_water_ increased but decreased with the increase of *T*_SP_. AWJM produces a high CRS state for all milled surfaces, whose values are very close in both longitudinal and transverse directions, from −292 MPa to −655 MPa, which indicates that the tool path has no effect whatsoever, equally to *P*_water_. However, increasing the *T*_SP_ and step-over distance favours the reduction of CRS. AWJM is a favourable process compared to conventional methods for milling INCONEL^®^ 718. Nevertheless, after manufacturing, the surfaces must be cleaned to remove embedded abrasive particles, generating up to 300% more *R*_a_ than CM. Srirangarajalu et al. [129] assessed the link between four critical process-independent variables: *T*_SP_, *AM*_FR_, *P*_water_ and gap distance (*G*_d_) to *R*_a_, kerf angle (*K*_θ_) and MRR when machining INCONEL^®^ 625 with AWJM. The response surface methodology-central composite design (RSM-CCD) method was used to perform the experimental interpretations. The influence of individual AWJM factors was determined using ANOVA analysis. SEM was used to inspect the surface morphology and erosion mechanisms. It was found that abrasive aqua jet pressure (AW_JP_) is the dominant factor in the responses. When the *P*_water_ = 300 MPa, *R*_a_ and *K*_θ_ reduced by 27.19 and 13.83%, respectively, MRR augmented by 23.71%. The desirability analysis was handled to optimise AWJM parameters of INCONEL^®^ 625. The optimal parameters obtained were *P*_water_ = 300 MPa, *T*_SP_ = 75 mm/min, *G*_d_ = 2.4 mm_,_ and *AM*_FR_ = 0.55 kg/min to minimise *R*_a_ and *K*_θ_ and maximise MRR. Vijayakumar et al. [130] determined the optimal parameter values, using a desirability analysis, to generate quality holes on INCONEL^®^ 625 by optimising abrasive water jet drilling (AWJD). *P*_water_, *G*_d_, *T*_SP_ and *AM*_FR_ of garnet and SiC were identified as potential process variables. *R*_a_, MRR, *C*_I_ and *C*_Y_ evaluated the quality of the hole surface. The influence of individual AWJM factors was determined using the ANOVA analysis. SEM and 3D images were used to analyse the hole’s surface topography on INCONEL^®^ 625 (Figure 39). *R*_a_, *C*_I_ and *C*_Y_ decreased by 27.35, 56.23 and 42.56%, and MRR increased by 32.83% when *P*_water_ = 300 MPa. According to a desirability analysis, the optimal machining parameters for numerous solutions are *P*_water_ = 300 MPa, *G*_d_ = 1 mm, *T*_SP_ = 72 mm/min and abrasive material constituted by 100% SiC. The ANOVA investigation revealed that *P*_water_ was the most contributed parameter, trailed by the abrasive material.

### 3.2. Surface Finish Processes

**Figure 40:**
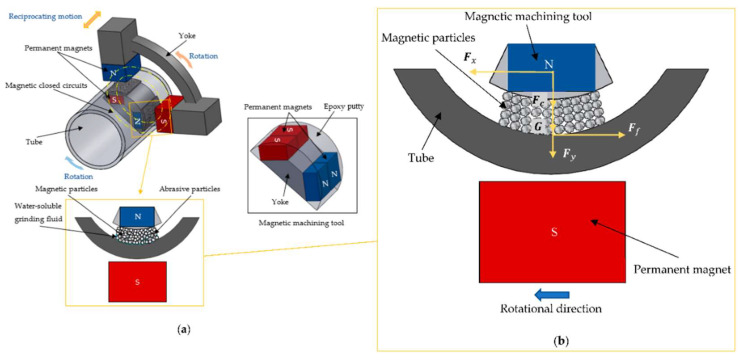
(**a**) Schematic of the magnetic abrasive finishing (similar to a honing operation) and the magnetic internal tool used, (**b**) zoomed-in schematic with force analysis model diagram (adapted from [132]).

Magnetic abrasive finishing (MAF, Figure 40) is a non-traditional post-abrasive technique to finish and polish hard-to-reach surfaces. It uses magnetic abrasive particles that are attracted to the workpiece surface and then moved back and forth using a magnetic field. This process can be further enhanced by coupling electrical pulse and ultrasonic treatment with MAF. This combination increases MRR, improves surface quality and reduces TW [131]. 

The coupling electrical pulse and ultrasonic treatment (CEPUT), proposed and developed by Wang et al. [133], is a non-traditional machining technique that involves applying high-frequency electrical pulses and ultrasonic waves to the workpiece, which removes material through electrochemical reactions and cavitation effects. The combined effect of electrical and ultrasonic energy can enhance the workpiece’s machining rate and surface finish, making it a promising candidate for various applications. Ultrasonic-assisted abrasive belt grinding (UAABG) is another non-traditional finishing technique that employs ultrasonic vibrations to enhance grinding [134]. The ultrasonic vibrations help to break down the abrasive particles and improve their cutting ability, resulting in increased MRR and improved surface finish. Robotic abrasive belt grinding (RABG) is a highly automated process that utilises robots [135] to perform abrasive belt grinding on workpieces. This technique offers precise control over the grinding process [136], ensuring consistent results and reducing operator fatigue. Additionally, using robots eliminates the need for manual labour and reduces the risk of injury.

**Figure 41:**
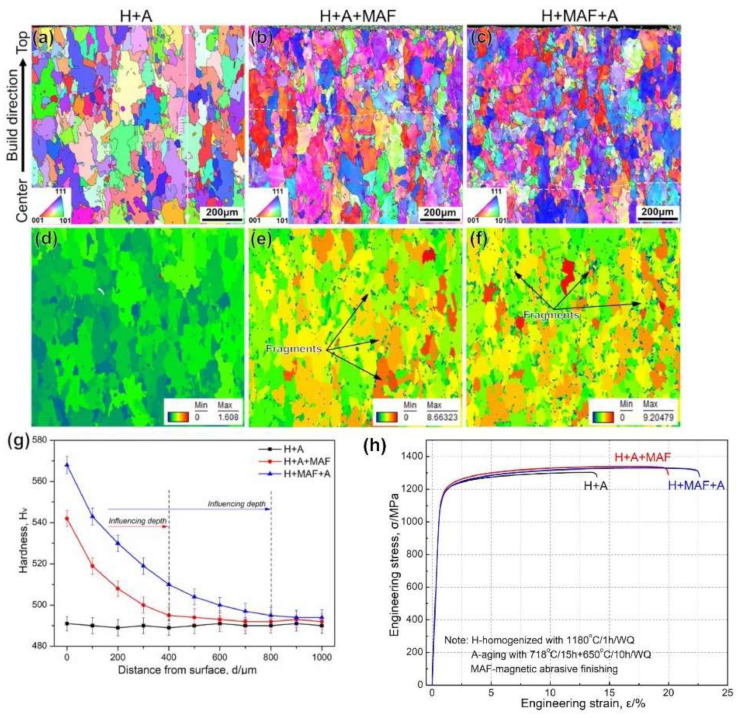
Microstructural characteristics and hardness variations from the surface to the core across the thickness of the specimens were investigated under various heat treatment (HT) and magnetic abrasive finishing (MAF) conditions: (**a**–**c**) orientation maps obtained through electron back-scattered diffraction (EBSD) observations, (**d**–**f**) corresponding grain orientation spread (GOS) maps derived from EBSD observations, (**g**) hardness progression from the surface, and (**h**) engineering stress–strain curves representing samples subjected to different post-processing conditions (adapted from [99]).

Li et al. [99] employed the hybrid post-processing techniques of MAF and subsequent post-heat treatment (HT) on LPBF-manufactured INCONEL^®^ 718. MAF demonstrated its influence on surface integrity, resulting in reduced *R*_a_ and MRR for INCONEL^®^ 718 (Figure 41a–g). The mechanical properties of AMed INCONEL^®^ 718 were enhanced through the combined application of MAF and HT (Figure 41h). A comparison between homogenized and aged (H + A) samples and those subjected to single HT post-processing revealed that the HT + A+ MAF specimen exhibited superior mechanical properties, achieving *σ*_y_ = 1151 MPa, *σ*_u_ = 1339 MPa, *ε*_u_ = 19.5% and a static toughness (*U*_0T_) of 254.3 MJ/m^3^. The finishing process reduced *R*_a_ to 0.15 μm after 180 min, inducing a shift from residual tensile stresses to compressive stresses in the surface layer of the AMed samples. This transformation increased crack growth resistance and ductility.

**Figure 42:**
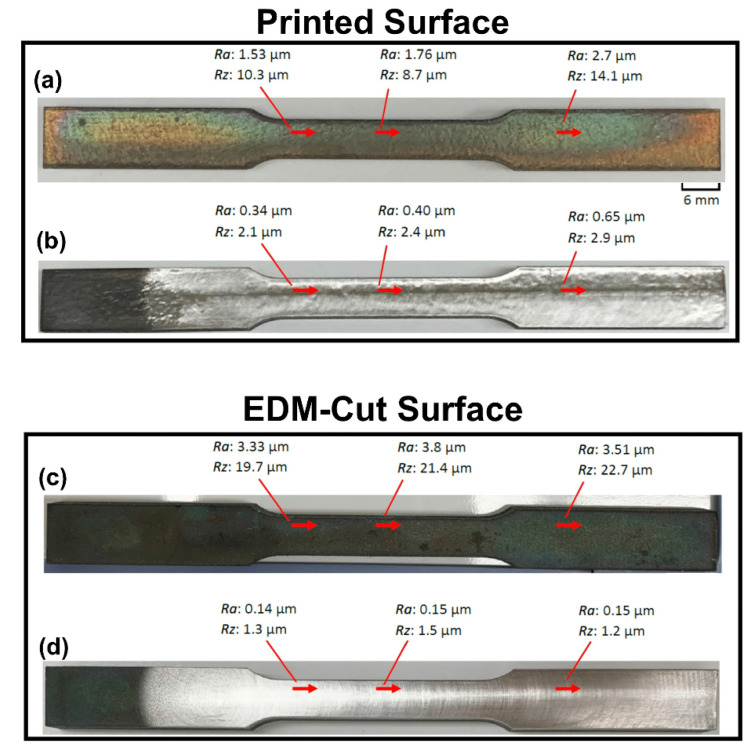
Visual representations of specimens subjected to complete heat treatment prior to magnetic abrasive finishing (MAF) (heat treatment (HT) + A) and subsequent to the MAF process (HT + A + MAF): (**a**) surface appearance pre-MAF and (**b**) post-MAF; (**c**) surface section following electrical discharge machining (EDM) pre-MAF and (**d**) post-MAF [137].

Zhao et al. [137] investigated the effects of different combinations of MAF and HT steps on the microstructure–property relationships of INCONEL^®^ 718 components made by LPBF. A comparison was made with tensile test samples fabricated by EDM (Figure 42). The MAF process can reduce the *R*_a_ of as-built and HT INCONEL^®^ 718 fabricated by LPBF. It was demonstrated that the printed tensile samples with HT + A + MAF had a better average *R*_a_ = 0.46 μm than HT + A with an average *R*_a_ = 2.0 μm. The same trend is visible throughout the EDM-cut tensile samples, having *R*_a_ = 0.15 μm with H + A + MAF, compared to *R*_a_ = 3.55 μm with HT + A. In the tensile tests, specimens with HT + A obtained *σ*_y_ = 1142.8 ± 2.1 MPa, *σ*_u_ = 1304 ± 3.2 MPa and *ε*_u_ = 14.0 ± 0.5%, specimens with HT + A + MAF obtained *σ*_y_=1152.1 ± 1.5 MPa, *σ*_u_=1340.4 ± 2.3 MPa and *ε*_u_=19.8 ± 0.5% and, finally, specimens with HT + MAF + A obtained *σ*_y_=1148.6 ± 1.8 MPa, *σ*_u_=1332.3 ± 2.6 MPa and *ε*_u_=22.7 ± 0.3%. These mechanical properties enhance the improvement made due to MAF, and applying it before or after the ageing process has different benefits.

**Figure 43:**
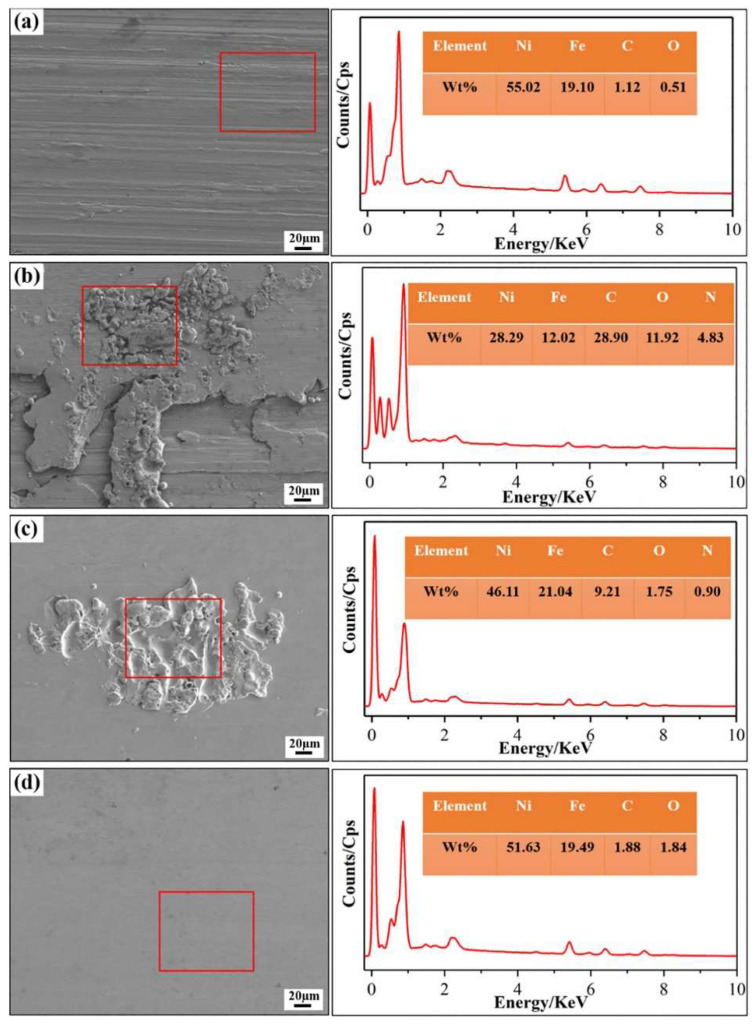
Surface morphology and elements of samples treated with different conditions (**a**) untreated, (**b**) treated with H_2_O, (**c**) treated with *wt* % = 20% emulsion, (**d**) treated with *wt* % = 40% emulsion [133].

Wang et al. [133] developed and applied a novel water-in-oil emulsion with an element weight percentage (*wt* %) of 40% H_2_O to INCONEL^®^ 718 CEPUT. After the treatment (Figure 43), a strengthening layer of about 450 μm was produced on the surface, with 503.8 HV surface hardness. *R*_a_ was enhanced to 0.042 μm, strengthening the surface of INCONEL^®^ 718. Also, the wear resistance of the metal surface is improved, and the sample surface’s average friction coefficient (*μ*_e_) is reduced by 22.3%. This decrease in friction coefficient leads to a reduction of 43.2% in wear compared to before the treatment.

**Figure 44:**
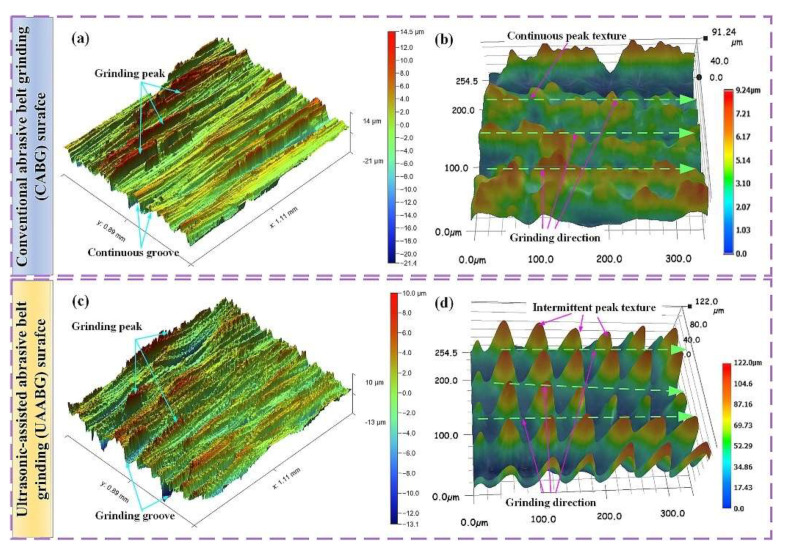
Grinding surface texture. (**a**) Conventional abrasive belt grinding (CABG) surface; (**b**) magnified view of CABG surface; (**c**) ultrasonic-assisted abrasive belt grinding (UAABG) surface; (**d**) magnified view of UAABG surface [134].

Li et al. [134] proposed a novel anti-fatigue grinding strategy, which enhances the fatigue performance and failure mechanism of UAABG applied to INCONEL^®^ 718. A comparison with conventional abrasive belt grinding (CABG) was performed. UAABG can reduce *R*_a_, increase surface CRS and microhardness and promote the formation of a uniform surface grain deformation layer compared to CABG (Figure 44). The fatigue performance of INCONEL^®^ 718 was improved between 14.3–74.8%, and fatigue life samples increased with the increase in line speed and the decrease in feed speed. The surface fatigue performance is the most sensitive to the feed speed. Nonetheless, precise regulation and control of service performance remains a major critical problem in the UAABG technology.

**Figure 45:**
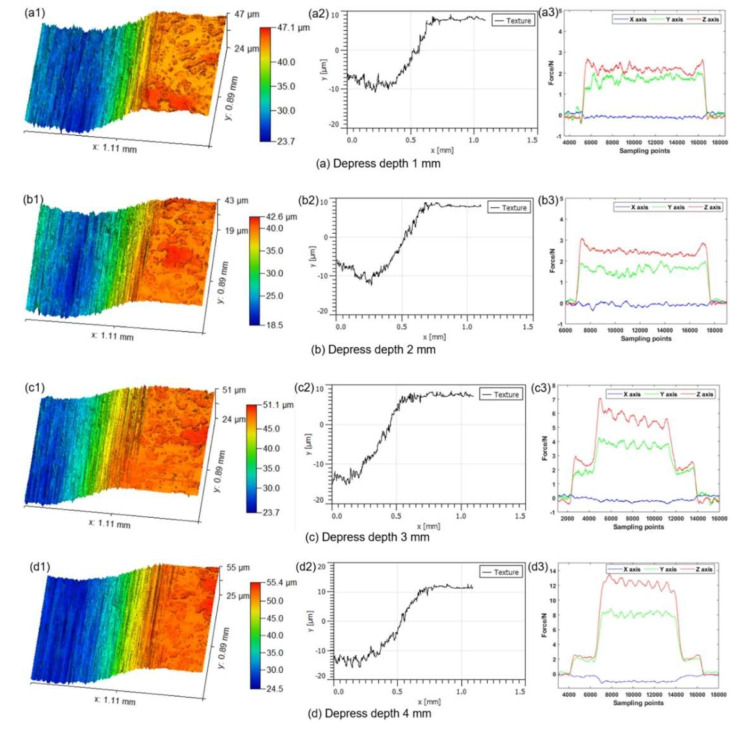
Depth and force of different grinding depress depth under up-grinding conditions (**a1**–**d1**) 3D texture graphs, (**a2**–**d2**) cross-sectional profile of 2D texture, seen from Y-axis, (**a3**–**d3**) grinding forces according to X, Y and Z-axis [136].

**Figure 46:**
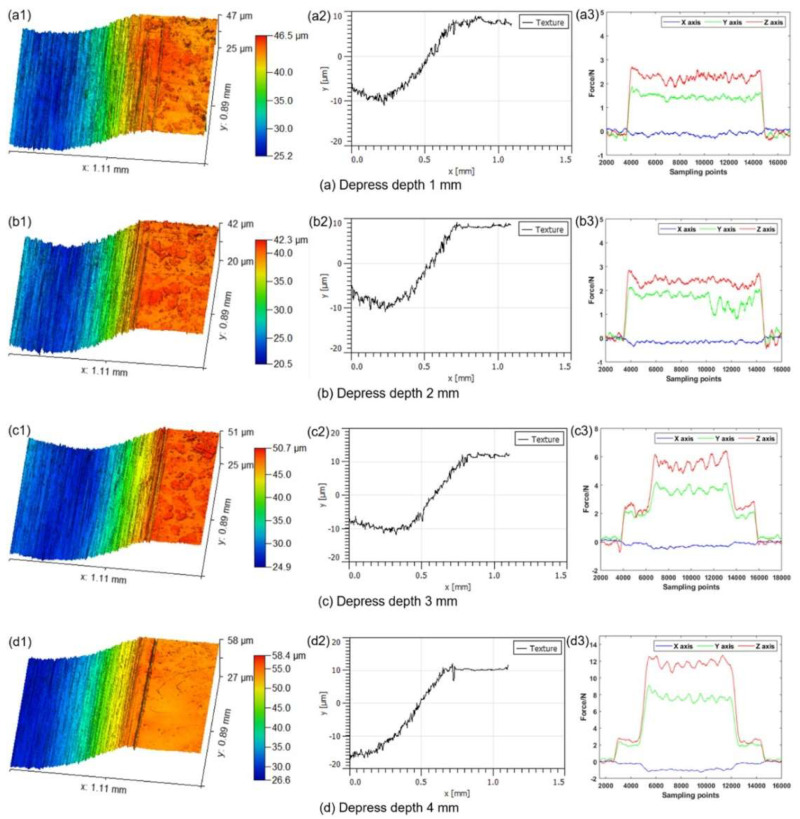
Depth and force of different grinding depress depth under down-grinding conditions (**a1**–**d1**) 3D texture graphs, (**a2**–**d2**) cross-sectional profile of 2D texture, seen from Y-axis, (**a3**–**d3**) grinding forces according to X, Y and Z-axis [136].

Song et al. [136] investigated the relationship between the grinding force and depth in the RABG, which was analysed in detail. The robot machining has an established error model considering the deformation of the grinding head when grinding INCONEL^®^ 718. With the increase in grinding depth by 52.94%, the grinding force shows an irregular increasing trend between 344.44 and 445.45%. This phenomenon happens because when the grinding depth is greater than 3 mm, the feed direction force and the normal force lead to prominent secondary pressure peaks at the start and end of grinding, somewhat unseen in previous studies. Analysis of force-depth, down-grinding and up-grinding of robotic abrasive belt grinding demonstrates that the grinding force ratio (*Y*/*Z*) decreases with the increase in the depress depth. For the down-grinding, *Y*/*Z* = 0.668, which is smaller than *Y*/*Z* = 0.724 for the up-grinding, considering the depress depth of 4 mm. Down-milling produced the best results, as depicted in Figure 45 vs. Figure 46, although depending on the application, it is recommended to choose between the two grinding methods.

## 4. Discussion

After the discussion on INCONEL^®^ machinability throughout this paper, a SWOT analysis was performed to discuss perceptions of the INCONEL^®^ machinability among the various manufacturing processes addressed. The INCONEL^®^ machinability analysis is divided into ECM (Table 1), EDM (Table 2), AM and the allied traditional processes (Table 3), TAM, USM and the allied traditional processes (Table 4), LBM and LDM (Table 5), AWJM (Table 6) and non-conventional surface finishing processes (Table 7).

## 5. Conclusions

In this paper, a comprehensive review of the non-conventional machining process applied to INCONEL^®^ alloys, namely 718 and 625, was given to the readers. At first glance, the subsequent research verified that more novel techniques are yet to be intensely exploited to effectively assess how further it can enhance INCONEL^®^ alloy manufacturing, juxtaposed with CM. Nevertheless, non-conventional INCONEL^®^ machining processes have characteristics that can effectively increase the mechanical properties of the produced components; for instance, some of the addressed ones do not need tool–workpiece contact, posing significant advantages over CM. The findings of this paper are presented below.

- **Enhanced Mechanical Properties:** Non-conventional INCONEL^®^ machining processes prove effective in enhancing the mechanical properties of manufactured components;
- **Diverse Techniques with Unique Advantages:** Each addressed technique offers unique advantages along with inherent disadvantages, providing a range of options for manufacturers;
- **Variability in Results:** Variability in input parameters across studies results in differing outcomes, emphasizing the need for a more generic understanding of each process;
- **EDM and Variants:** Essential in modern manufacturing, EDM and its variants contribute to increased productivity but require careful consideration of parameters;
- **Revolutionary Role of AM Techniques:** AM techniques revolutionize manufacturing, producing customized and complex parts with high precision, especially when combined with traditional processes;
- **Challenges in AM for INCONEL^®^ 625:** High residual stress, anisotropies and non-equilibrium solidification highlight the immaturity and insufficiency of AM parameters for INCONEL^®^ alloys;
- **Improvement and Competitiveness of TAM and USM:** TAM and USM techniques show promise in improving tool life, surface quality and productivity and emerge as competitive processes, but ongoing research and development are necessary for performance optimization and reliability;
- **Innovative LBM and LDM:** LBM and LDM excel in machining complex designs and drilling precise holes, requiring careful consideration of parameters and ongoing optimizations;
- **WJM and AWJM Dependence on Parameters:** AWJM are non-contact processes highly dependent on optimizing process parameters and material properties;
- **Need for Future Optimizations:** Despite being known processes, AWJM require future optimizations to comprehend inherent mechanisms and improve efficiency for various applications;
- **Potential of Surface Finishing Techniques:** The addressed surface finishing non-traditional machining techniques show potential in improving productivity and quality, yet further research is needed for optimization;
- **Skill Enhancement and Knowledge:** The work contributes to improving practitioners’ skills and knowledge, addressing challenges in each process, and encouraging a shift from conventional to more evolved techniques suitable for advanced materials like INCONEL^®^ alloys;
- **Equipment Cost and Industrial Performance:** Despite high equipment costs, wider adoption of unconventional equipment is crucial for achieving industrial performance, potentially leading to reduced prices and improved product quality;
- **Suitability for High-Performance Materials:** Unconventional processes are particularly suitable for high-performance materials, but training and expertise matching the sophistication of the equipment are crucial for successful implementation.

This work also aims to contribute to improving the practitioners’ skills, increasing knowledge around the usual difficulties of each process and encouraging the change from more conventional and less effective processes to more evolved processes, which can meet the ambition of designers and the characteristics of some of the most evolved materials, like the INCONEL alloys. Despite the high prices of unconventional equipment, they are essential to achieve the industrial performance required by the most daring designers. The larger scale adoption of this equipment will reduce the price, which will translate into greater ease of acquisition, raising the level of quality and accuracy of the manufactured products. Furthermore, unconventional processes are usually more suitable for high-performance materials. However, training and expertise appropriate to the sophistication presented by this type of equipment is crucial.

## Figures and Tables

**Table 1:** SWOT analysis of INCONEL^®^ machinability with ECM.

	Positive Factors	Negative Factors
**Internal factors**	**Strengths** **Precision Machining**: The anodic metal dissolution process offers great atomic precision, producing complex shapes and intricate details with high MRR. **Versatility**: It is suitable for machining various materials, including hard and brittle ones like INCONEL^®^ alloys, making it versatile for use in industries where precision and accuracy are paramount. There is no contact between the tool and the workpiece, and it does not produce heat; thus, the machined parts are not distorted. **Specialized Variants**: The existence of specialized variants like WECM, ECMG and ECD demonstrates the adaptability of ECM to address specific machining needs. **Optimization Techniques**: Research efforts showcase optimization techniques such as Taguchi approaches and enhanced tool designs, leading to improved machining characteristics and efficiency.	**Weakness** **Corrosion Resistance**: The corrosion resistance of INCONEL^®^ alloys may hinder the ECM process without a proper electrolyte medium. **SR**: Uneven bulges and a rough surface may develop in ECM, especially when dealing with modified or doped INCONEL^®^ alloys, as observed in the study by Ren et al., where the passivation film was removed, and the dissolution behaviour became uneven.
**External factors**	**Opportunities** **Enhanced Tool Design**: Ongoing research on enhanced tool designs provides opportunities to further improve the efficiency and effectiveness of ECM processes, especially for rough machining stages. **Material Modification**: Studies on modified or doped INCONEL^®^ alloys open avenues for understanding and improving the electrochemical dissolution behaviour, potentially leading to enhanced performance in ECM. **Hybrid Techniques**: Introducing hybrid machining techniques, such as HWECDM, demonstrates opportunities for innovation and improved efficiency, especially for machining large-thickness hard metal materials. **Corrosion Resistance**: Using electrolyte mediums like citric acid (Cit^3−^ or C_6_H_5_O_7_^3−^) or potassium citrate (C_6_H_5_K_3_O_7_) can enhance INCONEL^®^ ECM.	**Threats** **Challenges in Transport**: The intricate transport of electrolytic products in confined machining gaps poses a challenge, indicating potential difficulties in machining large-thickness workpieces using conventional ECM techniques. **Material Specificity**: The suitability of ECM for INCONEL^®^ alloys may limit its applicability to a broader range of materials, potentially restricting its usage in industries where diverse materials need precise machining. **Other Threats**: Associated cost, specialized personnel and process monitoring.

**Table 2:** SWOT analysis of INCONEL^®^ machinability with EDM.

	Positive Factors	Negative Factors
**Internal factors**	**Strengths** **High Precision Machining**: INCONEL^®^ EDM offers high precision in machining intricate and delicate parts, making it suitable for aerospace, automotive, medical and electronics applications. **Versatility**: EDM and its variants are versatile and capable of machining hardened metals and handling complex geometries that may be challenging for conventional machining methods. No contact is needed between the tool and workpiece. **Variants for Specific Applications**: The existence of variants like WEDM, MSEAM and HF-EDAM allows for tailored solutions to specific manufacturing requirements, offering flexibility and adaptability. **Optimization Techniques**: They demonstrate using optimization techniques like DOE and Taguchi methods to determine optimal parameters, maximize MRR and minimize *R*_a_ for better machining performance.	**Weakness** **Surface Quality Concerns**: Surface quality achieved through EDM processes, particularly WEDM, may be lower than alternative manufacturing processes, leading to potential concerns about surface integrity and fatigue life. **Limited Material Applicability**: While effective for INCONEL^®^ alloys, EDM may not be universally suitable for all materials, limiting its range of application in industries where diverse materials need precise machining. **Other weaknesses** are that it is environmentally sustainable, generates flammable gases and a characteristic white layer, and MRR and Ra parameters directly affect HAZ. Novel academic solutions take time to be applied to the industry.
**External factors**	**Opportunities** **Advanced Tool and Process Development**: Ongoing research in servo gap control mechanisms, magnetic levitation and novel electrode materials provide opportunities for advanced tool and process development, potentially improving efficiency, speed and SR. The cryogenically cooled electrode provides better machining performance than ordinary wire. **Hybrid Machining Techniques**: The exploration of hybrid processes, such as HF-EDAM, combining EDM with milling, showcases opportunities for innovation, offering high-quality and efficient machining of INCONEL^®^ alloys.	**Threats** **Competitive Alternative Processes**: EDM may face competition from alternative manufacturing processes in terms of SR, stress influence and fatigue life, posing a threat to its widespread adoption. **Challenges in Thermal Deformation**: Challenges in managing thermal deformation during WEDM indicate potential difficulties in achieving optimal results within typical parametric ranges. **Other Threats**: Associated cost, specialized personnel and process monitoring.

**Table 3:** SWOT analysis of INCONEL^®^ machinability with AM and the allied traditional processes.

	Positive Factors	Negative Factors
**Internal factors**	**Strengths** **Complex Geometries and Customization**: INCONEL^®^ AM processes enable high-precision production of complex geometries and customized products, which is particularly beneficial for industries requiring intricate and mission-critical components. **Reduced Debris Production:** AM is recognized for its sustainability-friendly approach, significantly reducing debris production and chipping during manufacturing and aligning with the growing emphasis on environmentally conscious practices. **Versatility in AM Techniques**: Techniques such as LPBF, DED and WAAM provide versatility regarding feedstock type, energy source and processing medium. This adaptability allows for a wide range of applications and customization possibilities. Moreover, the created anisotropy enhances machinability in specific directions.	**Weakness** **Post-Deposition Effects**: While WAAM offers high deposition rates, it has undesirable effects like higher dilution, thermal distortion and a more significant HAZ. Additionally, the Laves phase in INCONEL^®^ 718 microstructure may require modified post-deposition HT. **Surface Quality Challenges**: Certain AM methods, including WAAM, may face challenges related to surface quality. The need for post-processing steps to achieve the desired surface finish may be a drawback. **Other weaknesses**: Parameter definition is very immature due to complicated problems such as metallurgical, physical, chemical and thermal coupling and the subsequent relationship among them; novel academic solutions take time to be applied to the industry and highly energetic consumption (AM alone).
**External factors**	**Opportunities** **Advanced Cooling and Lubrication Techniques**: Machining characteristics can be improved through sustainable cooling conditions. Advanced cooling and lubrication techniques can enhance TL, reduce TW and improve SR. **Hybrid Manufacturing Processes**: The integration of AM with traditional processes offers opportunities to leverage the strengths of both methods. This hybrid approach can lead to improved efficiency and part quality. With technological advances and newer scientific investigations, AM can see weaknesses and problems resolved in the long term.	**Threats** **Competition from CM**: Despite its advantages, AM may face competition from well-established conventional processes, especially for specific applications. Traditional methods like drilling may still offer surface finish and efficiency advantages. **Material-Specific Challenges**: The use of INCONEL^®^ alloys in AM processes may pose challenges specific to the material, such as thermal deformation, post-deposition effects and the need for optimized heat treatments. Addressing these challenges is crucial for broader adoption.

**Table 4:** SWOT analysis of INCONEL^®^ machinability with TAM, USM, and the allied traditional processes.

	Positive Factors	Negative Factors
**Internal factors**	**Strengths** **Enhanced Efficiency**: TAM improves machining efficiency by softening the workpiece. Combining heat sources with traditional processes enhances INCONEL^®^ alloy machinability. This results in reduced *F*_c_ and improved MRR. **Improved Surface Finish**: TAM processes contribute to smoother and more precise cuts, leading to a better *R*a, particularly evident in studies involving INCONEL^®^ 718, where TAM shows a significant reduction in *R*_a_ compared to CM. **Reduced TW**: TAM processes exhibit reduced tool wear and subsurface damage, positively impacting TL and overall tool performance during machining. **Tailored Material Properties**: TAM allows for customising material properties, such as surface hardness and wear resistance. Combining heat sources with traditional processes enhances INCONEL^®^ alloys machinability (TAM). Using an optimized heat shield may enhance the performance of these processes (TAM). Combining ultrasonic systems with traditional processes improves machinability through better and higher MRR values (USM).	**Weakness** **Complex Process Integration**: Implementing TAM requires additional energy sources and complex machinery, which may lead to higher initial setup costs. Integrating lasers, plasma arcs or induction coils adds complexity to the machining system. Heat sources affect INCONEL^®^ alloys’ surface and lower the tool’s superficial hardness if a heat shield (TAM) is not used. **Precision Challenges**: Achieving optimal results with TAM processes demands precise control over various parameters, including laser power, cutting speeds and feed rates. Variations in these parameters may affect the quality and consistency of machining outcomes. **Material-Specific Optimization**: The effectiveness of TAM processes may vary depending on the material being machined. While studies show positive results for INCONEL^®^ alloys, the applicability to other materials might require specific process adjustments. Ultrasonic systems tend to increase TW, leading to a poorer TL (USM).
**External factors**	**Opportunities** **Broad Material Applicability**: TAM processes have shown promise in machining challenging materials like INCONEL^®^ alloys. There is an opportunity to explore and optimize these processes for a broader range of materials, expanding their applicability. **Technological Advancements**: Continuous advancements in TAM technologies, including improvements in laser technology and plasma systems, present opportunities for enhanced precision, efficiency and reduced costs. In the mid-term, these hybrid processes (TAM and USM) are the best to adopt regarding the remaining addressed non-conventional processes. **Environmental Sustainability**: TAM processes can be positioned as environmentally sustainable options if energy-efficient sources are employed. Using lasers and induction for localized heating may contribute to reduced energy consumption compared to conventional methods.	**Threats** **High Initial Investment**: The initial cost of acquiring and setting up TAM equipment, especially for advanced processes like LBP, may pose a barrier to adoption for some manufacturers. **Competitive Traditional Machining**: Traditional machining methods continue to be widely adopted, and advancements in cutting tools and techniques might pose competition to adopting TAM processes. With technological advances, these hybrid processes may be rapidly surpassed by purely non-conventional processes. **Skill Requirements**: Operating TAM processes effectively requires skilled personnel with expertise in CM and the additional technologies involved. The shortage of skilled operators might impede the widespread adoption of TAM.

**Table 5:** SWOT analysis of INCONEL^®^ machinability with LBM and LDM.

	Positive Factors	Negative Factors
**Internal factors**	**Strengths** **High Precision and Accuracy**: LBM and LDM provide precise and accurate machining, making them suitable for applications where intricate designs and tight tolerances are crucial. **Versatility in Material Machining**: LBM and LDM showcase the ability to machine a wide range of materials, including metals like INCONEL^®^ alloys, ceramics, plastics and composites. This versatility enhances their applicability across various industries. **Complex Geometry Capability**: These processes can handle complex and intricate geometries, making them valuable for manufacturing components with intricate designs or structures that are challenging to achieve through traditional machining methods. **Non-contact Machining**: LBM and LDM are non-contact machining techniques, reducing the risk of tool wear and minimizing the chances of contamination, which is particularly advantageous for machining delicate parts and maintaining material integrity.	**Weakness** **Thermal Stresses and Residual Stresses**: The main drawback of LBM and LDM is the generation of thermal stresses and residual stresses during the machining process. These stresses can potentially affect the machined components’ structural integrity and dimensional stability. **Surface Quality Concerns**: Achieving optimal surface quality is a challenge due to the thermal effects involved in the process. Issues such as surface defects and thermal damage may arise, impacting the final finish of the machined surfaces. Novel academic solutions take time to be applied to the industry. Highly energetic consumption.
**External factors**	**Opportunities** **Parameters Optimization**: Continuous research and optimization of laser parameters provide an opportunity to enhance the efficiency and effectiveness of LBM and LDM. Identifying optimal configurations, such as pulse frequency, scan speed and laser intensity, can improve machining results. **Advanced Process Enhancements**: Exploring advanced machining procedures, like those involving TGRA, offers opportunities to refine the process factors to achieve better MRR and SR. **Increased Application Scope**: As technology advances, there is an opportunity to expand the application scope of LBM and LDM to a broader range of industries beyond aerospace and automotive. This could involve adapting the processes for diverse materials and component types.	**Threats** **Competition from Traditional Methods**: Despite their limitations, traditional machining techniques continue to be widely adopted. LBM and LDM may face competition from established methods, especially in industries where transitioning to non-traditional methods is challenging. **Challenges in Thermal Management**: Overcoming challenges related to thermal stresses and achieving better control over thermal effects during machining is critical. Failure to address these challenges may limit the acceptance of LBM and LDM in specific applications. **Cost Considerations**: The initial setup and operational costs associated with LBM and LDM equipment can be high. Cost considerations might pose a threat, especially in industries where budget constraints are significant.

**Table 6:** SWOT analysis about INCONEL^®^ machinability with AWJM.

	Positive Factors	Negative Factors
**Internal factors**	**Strengths** **Material Compatibility**: WJM effectively machines ductile and sensitive materials like INCONEL^®^ alloys, providing a versatile solution for challenging materials. It does not produce heat; thus, the machined parts are not distorted. **SR**: WJM, especially with abrasives AWJM, can produce high-quality surfaces with minimal thermal warping and material distortion. This is crucial for maintaining the integrity of sensitive materials like INCONEL^®^. **Environmental Friendliness**: WJM and AWJM are considered environmentally friendly, as they do not produce hazardous waste or emissions, aligning with the increasing emphasis on sustainable manufacturing. **High Precision and Tolerances**: The processes offer high precision and accuracy, allowing for producing parts with tolerances as low as 10 μm. This is essential for applications where precision is a critical factor.	**Weakness** **Surface Hardness:** AWJM may exhibit lower surface hardness values than alternative manufacturing processes, a limitation for applications where high hardness is a critical requirement. **Fatigue Limit**: The relatively shallow penetration depth of AWJM may result in a lower fatigue limit. This can be a weakness, especially in applications where components are subjected to cyclic loading. **Post-Machining Cleaning Requirement**: Surfaces machined with AWJM may require additional cleaning to remove embedded abrasive particles. Failure to do so can result in higher surface roughness compared to CM. AWJM produces a “sludge” due to the abrasive powder it creates.
**External factors**	**Opportunities** **Technological Advancements**: Ongoing advancements in waterjet machining technologies, including nozzle design, abrasive materials and control systems, present opportunities for improving efficiency and addressing weaknesses. **Increased Acceptance in Aerospace and Specialty Applications**: As technology and processes mature, there is an opportunity for broader acceptance of water-jet machining in aerospace and other speciality applications where INCONEL^®^ alloys are prevalent.	**Threats** **Competition from Alternative Machining Techniques**: Water-jet machining faces competition from alternative machining methods, including CM and other non-traditional techniques. The choice of the optimal technique may threaten the adoption of WJM for INCONEL^®^ alloys. **Initial Investment Costs:** The initial costs associated with acquiring and setting up water-jet machining equipment, especially AWJM, may be relatively high. This could limit adoption, particularly for smaller manufacturers or those with budget constraints.

**Table 7:** SWOT analysis of INCONEL® machinability with non-conventional surface finish processes.

	Positive Factors	Negative Factors
**Internal factors**	**Strengths** **Versatility of Processes**: The variety of surface finish processes, including MAF, CEPUT, UAABG, RABG and post-heat treatment (HT), provide various options to cater to diverse finishing requirements for INCONEL^®^ alloys. **Surface Quality Enhancement**: These surface finishing techniques, when appropriately applied, demonstrate the capability to enhance surface quality by reducing SR, improving microhardness, and promoting the formation of uniform surface grain deformation layers. **Mechanical Property Improvement**: The combination of certain processes, such as MAF and subsequent HT, has shown the potential to improve the mechanical properties of INCONEL^®^ 718, leading to enhanced strength, ductility and crack growth resistance. **Automated and Precise Control**: RABG offers automated precision, ensuring consistent results and reducing the risk of human error. This is crucial for maintaining quality standards. Enhancement of the mechanical properties, namely surface hardness.	**Weakness** **Process Sensitivity**: The effectiveness of certain processes, like UAABG, can be sensitive to specific parameters, such as line speed and feed speed. Precise regulation and control may be challenging, potentially limiting the widespread application of these techniques. **Complexity and Integration**: Integrating multiple processes, as seen in hybrid approaches like MAF and subsequent post-HT, can introduce complexity into the finishing process. This complexity may require careful management and control. Not-so-established processes need precise regulation and control of service performance.
**External factors**	**Opportunities** **Technological Advancements**: Ongoing advancements in surface finish technologies, such as improvements in abrasive materials, tool designs and control systems, present opportunities to enhance the efficiency and effectiveness of the finishing processes for INCONEL^®^ alloys. **Broader Acceptance in Additive Manufacturing**: As the use of additive manufacturing, like LPBF, continues to grow, there is an opportunity for surface finish processes to play a vital role in enhancing the properties of additively manufactured components, specifically those made from INCONEL^®^ alloys.	**Threats** **Competition from Traditional Methods**: Surface finish processes face competition from traditional finishing methods. The choice between conventional abrasive processes and non-traditional techniques may influence the adoption of these newer methods for INCONEL^®^ alloys. **Regulatory and Environmental Constraints**: Stringent regulations and environmental concerns related to using certain abrasive materials or chemicals in the finishing processes may pose threats. Adherence to environmental standards may impact the choice of finishing techniques.

## Data Availability

No new data were created.

## References

[1] Weber J.H., Banerjee M.K. (2019). Nickel and Nickel Alloys: An Overview. Reference Module in Materials Science and Materials Engineering.

[2] Ding J., Xue S., Shang Z., Li J., Zhang Y., Su R., Niu T., Wang H., Zhang X. (2021). Characterization of precipitation in gradient Inconel 718 superalloy. Mater. Sci. Eng. A.

[3] Chen Z., Song Soh G., Zhou K. (2023). Chapter 6—Wire Arc Additive Manufacturing: Systems, Microstructure, Defects, Quality Control, and Modelling. Additive Manufacturing: Materials, Functionalities and Applications.

[4] Campbell F.C., Campbell F.C. (2006). Chapter 6—Superalloys. Manufacturing Technology for Aerospace Structural Materials.

[5] Liu X., Fan J., Zhang P., Cao K., Wang Z., Chen F., Liu D., Tang B., Kou H., Li J. (2023). Influence of heat treatment on Inconel 625 superalloy sheet: Carbides, γ″, δ phase precipitation and tensile deformation behavior. J. Alloys Compd..

[6] Kassner M.E., Kassner M.E. (2015). Chapter 11—γ/γ′ Nickel-Based Superalloys. Fundamentals of Creep in Metals and Alloys.

[7] Nomoto H., Tanuma T. (2022). 13—Development in materials for ultra-supercritical and advanced ultra-supercritical steam turbines. Advances in Steam Turbines for Modern Power Plants.

[8] Pedroso A.F.V., Sebbe N.P.V., Costa R.D.F.S., Barbosa M.L.S., Sales-Contini R.C.M., Silva F.J.G., Campilho R.D.S.G., De Jesus A.M.P. (2024). INCONEL® Alloy Machining and Tool Wear Finite Element Analysis Assessment: An Extended Review. J. Manuf. Mater. Process..

[9] Murty B.S., Yeh J.W., Ranganathan S., Bhattacharjee P.P., Murty B.S., Yeh J.W., Ranganathan S., Bhattacharjee P.P. (2019). 8—Special subgroups of high-entropy alloys. High-Entropy Alloys.

[10] De Bartolomeis A., Newman S.T., Jawahir I.S., Biermann D., Shokrani A. (2021). Future research directions in the machining of Inconel 718. J. Mater. Process. Technol..

[11] Andresen P.L., Féron D., Staehle R.W. (2016). 5—Understanding and predicting stress corrosion cracking (SCC) in hot water. Stress Corrosion Cracking of Nickel Based Alloys in Water-Cooled Nuclear Reactors.

[12] Madariaga A., Garay A., Esnaola J.A., Arrazola P.J., Linaza A. (2022). Effect of surface integrity generated by machining on isothermal low cycle fatigue performance of Inconel 718. Eng. Fail. Anal..

[13] Yang J., Wang S., Xu D., Guo Y., Yang C., Li Y. (2017). Effect of ammonium chloride on corrosion behavior of Ni-based alloys and stainless steel in supercritical water gasification process. Int. J. Hydrogen Energy.

[14] Chêne J., Féron D., Staehle R.W. (2016). 7—Stress corrosion cracking and hydrogen embrittlement. Stress Corrosion Cracking of Nickel Based Alloys in Water-cooled Nuclear Reactors.

[15] Frangini S., Paoletti C., Della Seta L. (2021). Corrosion of inconel alloys for application as inert anodes in low-temperature molten carbonate electrolysis processes. Int. J. Hydrogen Energy.

[16] Wei C., Wang Z., Chen J. (2022). Sulfuration corrosion failure analysis of Inconel 600 alloy heater sleeve in high-temperature flue gas. Eng. Fail. Anal..

[17] Yin M., Shao Y., Kang X., Long J., Zhang X. (2023). Fretting corrosion behavior of WC-10Co-4Cr coating on Inconel 690 alloy by HVOF thermal spraying. Tribol. Int..

[18] Zhang W., Xu Y., Shi Y., Su G., Gu Y., Volodymyr K. (2022). Intergranular corrosion characteristics of high-efficiency wire laser additive manufactured Inconel 625 alloys. Corros. Sci..

[19] Soundararajan C.K., Wang D., Vinogradov A. (2023). Effect of hydrogen on nanomechanical properties of Inconel 625 studied using in-situ electrochemical nanoindentation technique. J. Alloys Compd..

[20] Rodriguez D., Merwin A., Karmiol Z., Chidambaram D. (2017). Surface chemistry and corrosion behavior of Inconel 625 and 718 in subcritical, supercritical, and ultrasupercritical water. Appl. Surf. Sci..

[21] Anburaj R., Pradeep Kumar M. (2021). Experimental studies on cryogenic CO2 face milling of Inconel 625 superalloy. Mater. Manuf. Process..

[22] Li X., Liu X., Yue C., Liang S.Y., Wang L. (2022). Systematic review on tool breakage monitoring techniques in machining operations. Int. J. Mach. Tools Manuf..

[23] Polishetty A., Littlefair G., Gupta K., Pramanik A. (2021). Chapter 1—Advances in conventional machining processes for machinability enhancement of difficult-to-machine materials. Advanced Machining and Finishing.

[24] Weglowski M.S., Błacha S., Phillips A., Chaturvedi M. (2021). 6—Electron beam welding—Techniques and trends. Welding and Joining of Aerospace Materials.

[25] Minet K., Saharan A., Loesser A., Raitanen N., Froes F., Boyer R. (2019). 8—Superalloys, powders, process monitoring in additive manufacturing. Additive Manufacturing for the Aerospace Industry.

[26] Hosseini E., Popovich V.A. (2019). A review of mechanical properties of additively manufactured Inconel 718. Addit. Manuf..

[27] Wang Q., Ge S., Wu D., Ma H., Kang J., Liu M., Wang T., Narayanaswamy B., Su R. (2022). Evolution of microstructural characteristics during creep behavior of Inconel 718 alloy. Mater. Sci. Eng. A.

[28] Bhattacharyya B., Doloi B., Bhattacharyya B., Doloi B. (2020). Chapter Two—Classification of advanced machining technology. Modern Machining Technology.

[29] Wang Y.C., Wang L.Y., Zhang B., Song Z.M., Luo X.M., Zhang G.P. (2022). Building height-related creep properties of Inconel 718 superalloy fabricated by laser powder bed fusion. Mater. Sci. Eng. A.

[30] Kim K.-S., Kang T.-H., Kassner M.E., Son K.-T., Lee K.-A. (2020). High-temperature tensile and high cycle fatigue properties of inconel 625 alloy manufactured by laser powder bed fusion. Addit. Manuf..

[31] Kurniawan R., Park G.C., Park K.M., Zhen Y., Kwak Y.I., Kim M.C., Lee J.M., Ko T.J., Park C.-S. (2020). Machinability of modified Inconel 713C using a WC TiAlN-coated tool. J. Manuf. Process..

[32] Sebbe N.P.V., Fernandes F., Silva F.J.G., Sousa V.F.C., Sales-Contini R.C.M., Campilho R.D.S.G., Pedroso A.F.V. (2024). Wear Behavior Analysis of TiN/TiAlN Coated Tools in Milling of Inconel 718. Proceedings of the Flexible Automation and Intelligent Manufacturing: Establishing Bridges for More Sustainable Manufacturing Systems.

[33] Sousa V.F.C., Silva F.J.G., Alexandre R., Fecheira J.S., Silva F.P.N. (2021). Study of the wear behaviour of TiAlSiN and TiAlN PVD coated tools on milling operations of pre-hardened tool steel. Wear.

[34] Sousa V.F.C., Fernandes F., Silva F.J.G., Costa R.D.F.S., Sebbe N., Sales-Contini R.C.M. (2023). Wear Behavior Phenomena of TiN/TiAlN HiPIMS PVD-Coated Tools on Milling Inconel 718. Metals.

[35] Silva F.J.G., Sebbe N.P.V., Costa R.D.F.S., Pedroso A.F.V., Sales-Contini R.C.M., Barbosa M.L.S., Martinho R.P. (2024). Investigations on the Surface Integrity and Wear Mechanisms of TiAlYN-Coated Tools in Inconel 718 Milling Operations. Materials.

[36] Osmond L., Curtis D., Slatter T. (2021). Chip formation and wear mechanisms of SiAlON and whisker-reinforced ceramics when turning Inconel 718. Wear.

[37] (1993). Tool-Life Testing with Single-Point Turning Tools.

[38] Toubhans B., Fromentin G., Viprey F., Karaouni H., Dorlin T. (2020). Machinability of inconel 718 during turning: Cutting force model considering tool wear, influence on surface integrity. J. Mater. Process. Technol..

[39] Sousa V.F.C., Silva F.J.G., Fecheira J.S., Lopes H.M., Martinho R.P., Casais R.B., Ferreira L.P. (2020). Cutting Forces Assessment in CNC Machining Processes: A Critical Review. Sensors.

[40] Pleta A., Nithyanand G., Niaki F.A., Mears L. (2019). Identification of optimal machining parameters in trochoidal milling of Inconel 718 for minimal force and tool wear and investigation of corresponding effects on machining affected zone depth. J. Manuf. Process..

[41] (1989). Tool Life Testing in Milling—Part 2: End Milling.

[42] Suárez A., Veiga F., Polvorosa R., Artaza T., Holmberg J., De Lacalle L.N.L., Wretland A. (2019). Surface integrity and fatigue of non-conventional machined Alloy 718. J. Manuf. Process..

[43] Pedroso A.F.V., Sousa V.F.C., Sebbe N.P.V., Silva F.J.G., Campilho R.D.S.G., Sales-Contini R.C.M., Jesus A.M.P. (2023). A Comprehensive Review on the Conventional and Non-Conventional Machining and Tool-Wear Mechanisms of INCONEL®. Metals.

[44] Pedroso A.F.V., Sousa V.F.C., Sebbe N.P.V., Silva F.J.G., Campilho R.D.S.G., Sales-Contini R.C.M., Nogueira F.R. (2024). A Review of INCONEL® Alloy’s Non-conventional Machining Processes. Proceedings of the Flexible Automation and Intelligent Manufacturing: Establishing Bridges for More Sustainable Manufacturing Systems.

[45] Liao Y., Deschamps F., Loures E.d.F.R., Ramos L.F.P. (2017). Past, present and future of Industry 4.0—A systematic literature review and research agenda proposal. Int. J. Prod. Res..

[46] Azarian M., Yu H., Shiferaw A.T., Stevik T.K. (2023). Do We Perform Systematic Literature Review Right? A Scientific Mapping and Methodological Assessment. Logistics.

[47] Tóth Á., Suta A., Pimentel J., Argoti A. (2023). A comprehensive, semi-automated systematic literature review (SLR) design: Application to P-graph research with a focus on sustainability. J. Clean. Prod..

[48] Sharma P., Chakradhar D., Gupta K., Pramanik A. (2021). Chapter 10—Advancements in electrochemical machining. Advanced Machining and Finishing.

[49] Noor W.I., Saleh T., Saleh T., Mohamed Ali M.S., Takahata K. (2021). Chapter 13—Electrochemical machining for microfabrication. Micro Electro-Fabrication.

[50] Ranjan R., Rai R.S., Bajpai V., Gupta K., Pramanik A. (2021). Chapter 7—Advances in conventional and nonconventional high-speed machining. Advanced Machining and Finishing.

[51] Perveen A., Akar S., Saleh T., Mohamed Ali M.S., Takahata K. (2021). Chapter 12—Electrochemical discharge machining: Trends and development. Micro Electro-Fabrication.

[52] Manikandan N., Kumanan S., Sathiyanarayanan C. (2017). Multiple performance optimization of electrochemical drilling of Inconel 625 using Taguchi based Grey Relational Analysis. Eng. Sci. Technol. Int. J..

[53] Niu S., Qu N., Yue X., Li H. (2019). Effect of tool-sidewall outlet hole design on machining performance in electrochemical mill-grinding of Inconel 718. J. Manuf. Process..

[54] Wang J., Xu Z., Wang J., Zhu D. (2021). Anodic dissolution characteristics of Inconel 718 in C_6_H_5_K_3_O_7_ and NaNO_3_ solutions by pulse electrochemical machining. Corros. Sci..

[55] Kong W., Zeng Y., Liu Z., Hu X., Kong H. (2022). Helical wire electrochemical discharge machining on large-thickness Inconel 718 alloy in low-conductivity salt-glycol solution. Chin. J. Aeronaut..

[56] Ren M., Zhu D., Li Z., Lei G. (2023). Electrochemical dissolution behavior of phosphorus and boron doped Inconel 718 alloy in NaNO_3_ solution. J. Alloys Compd..

[57] Liu L., Thangaraj M., Karmiris-Obratański P., Zhou Y., Annamalai R., Machnik R., Elsheikh A., Markopoulos A.P. (2022). Optimization of Wire EDM Process Parameters on Cutting Inconel 718 Alloy with Zinc-Diffused Coating Brass Wire Electrode Using Taguchi-DEAR Technique. Coatings.

[58] Kliuev M., Florio K., Akbari M., Wegener K. (2019). Influence of energy fraction in EDM drilling of Inconel 718 by statistical analysis and finite element crater-modelling. J. Manuf. Process..

[59] Kathiresan M., Theerkka Tharisanan R., Pandiarajan P., Kumar K., Kakandikar G., Davim J.P. (2022). Chapter Seven—Computational analysis of provisional study on white layer properties by EDM vs. WEDM of aluminum metal matrix composites. Computational Intelligence in Manufacturing.

[60] Xu M., Wei R., Li C., Ko T.J. (2023). High-frequency electrical discharge assisted milling of Inconel 718 under copper-beryllium bundle electrodes. J. Manuf. Process..

[61] Yue X., Yang X. (2021). The role of discharge plasma on molten pool dynamics in EDM. J. Mater. Process. Technol..

[62] Sivaprakasam P., Hariharan P., Gowri S., Udaya Prakash J., Kumar K., Kakandikar G., Davim J.P. (2022). Chapter Six—Machining performance analysis of micro-ED milling process of titanium alloy (Ti-6Al-4V). Computational Intelligence in Manufacturing.

[63] Rahman M.A., Ahmed A., Mia M., Saleh T., Mohamed Ali M.S., Takahata K. (2021). Chapter 3—Trends in electrical discharge machining of Ti- and Ni-based superalloys: Macro-micro-compound arc/spark/melt process. Micro Electro-Fabrication.

[64] Goyal A. (2017). Investigation of material removal rate and surface roughness during wire electrical discharge machining (WEDM) of Inconel 625 super alloy by cryogenic treated tool electrode. J. King Saud Univ. Sci..

[65] Rahul, Datta S., Biswal B.B., Mahapatra S.S. (2019). Machinability analysis of Inconel 601, 625, 718 and 825 during electro-discharge machining: On evaluation of optimal parameters setting. Measurement.

[66] Zhang Y., Guo S., Zhang Z., Huang H., Li W., Zhang G., Huang Y. (2019). Simulation and experimental investigations of complex thermal deformation behavior of wire electrical discharge machining of the thin-walled component of Inconel 718. J. Mater. Process. Technol..

[67] Farooq M.U., Anwar S., Kumar M.S., AlFaify A., Ali M.A., Kumar R., Haber R. (2022). A Novel Flushing Mechanism to Minimize Roughness and Dimensional Errors during Wire Electric Discharge Machining of Complex Profiles on Inconel 718. Materials.

[68] Sharma D., Bhowmick A., Goyal A. (2022). Enhancing EDM performance characteristics of Inconel 625 superalloy using response surface methodology and ANFIS integrated approach. CIRP J. Manuf. Sci. Technol..

[69] Kumar D., Sisodiya M.S., Mandal D.K., Bajpai V. (2023). Maglev micro-EDM: Feasibility and performance on Inconel 625. CIRP J. Manuf. Sci. Technol..

[70] (2015). Standard Specification for Copper-Beryllium Alloy Plate, Sheet, Strip, and Rolled Bar..

[71] Mahbub M.R., Rashid A., Jahan M.P., Gupta K., Pramanik A. (2021). Chapter 8—Hybrid machining and finishing processes. Advanced Machining and Finishing.

[72] Lu C., Shi J. (2022). Simultaneous consideration of relative density, energy consumption, and build time for selective laser melting of Inconel 718: A multi-objective optimization study on process parameter selection. J. Clean. Prod..

[73] (2016). Standard Specification for Precipitation-Hardening and Cold Worked Nickel Alloy Bars, Forgings, and Forging Stock for Moderate or High Temperature Service.

[74] Yangfan W., Xizhang C., Chuanchu S. (2019). Microstructure and mechanical properties of Inconel 625 fabricated by wire-arc additive manufacturing. Surf. Coat. Technol..

[75] Guimarães R.P.M., Minkowitz L., Arneitz S., Sommitsch C., Giedenbacher J., Müller M., Huskic A., Wild N., Buzolin R.H., Meier B., Salunkhe S., Amancio-Filho S.T., Davim J.P. (2023). Chapter 1—Powder bed fusion processes: Main classes of alloys, current status, and technological trends. Advances in Metal Additive Manufacturing.

[76] Guimarães R.P.M., Pixner F., Enzinger N., Feliciano Belei C.A., Effertz P.d.S., Amancio-Filho S.T., Salunkhe S., Amancio-Filho S.T., Davim J.P. (2023). Chapter 2—Directed energy deposition processes and process design by artificial intelligence. Advances in Metal Additive Manufacturing.

[77] Sebbe N.P.V., Fernandes F., Sousa V.F.C., Silva F.J.G. (2022). Hybrid Manufacturing Processes Used in the Production of Complex Parts: A Comprehensive Review. Metals.

[78] Sanchez S., Smith P., Xu Z., Gaspard G., Hyde C.J., Wits W.W., Ashcroft I.A., Chen H., Clare A.T. (2021). Powder Bed Fusion of nickel-based superalloys: A review. Int. J. Mach. Tools Manuf..

[79] Vaezi M., Drescher P., Seitz H. (2020). Beamless Metal Additive Manufacturing. Materials.

[80] Avery D.Z., Rivera O.G., Mason C.J.T., Phillips B.J., Jordon J.B., Su J., Hardwick N., Allison P.G. (2018). Fatigue Behavior of Solid-State Additive Manufactured Inconel 625. JOM.

[81] Balbaa M., Mekhiel S., Elbestawi M., McIsaac J. (2020). On selective laser melting of Inconel 718: Densification, surface roughness, and residual stresses. Mater. Des..

[82] Elshalakany A.B., Abdel-Mottaleb M.M., Salunkhe S., Alqahtani B., Salunkhe S., Amancio-Filho S.T., Davim J.P. (2023). Chapter 7—Mechanical properties of titanium alloys additive manufacturing for biomedical applications. Advances in Metal Additive Manufacturing.

[83] Nguyen Q.B., Nai M.L.S., Zhu Z., Sun C.-N., Wei J., Zhou W. (2017). Characteristics of Inconel Powders for Powder-Bed Additive Manufacturing. Engineering.

[84] (2017). Standard Test Method for Apparent Density of Free-Flowing Metal Powders Using the Hall Flowmeter Funnel.

[85] Alonso U., Veiga F., Suárez A., Gil Del Val A. (2021). Characterization of Inconel 718® superalloy fabricated by wire Arc Additive Manufacturing: Effect on mechanical properties and machinability. J. Mater. Res. Technol..

[86] Jhavar S., Manjaiah M., Raghavendra K., Balashanmugam N., Davim J.P. (2021). Chapter 9—Wire arc additive manufacturing: Approaches and future prospects. Additive Manufacturing—A Tool for Industrial Revolution 4.0.

[87] Seow C.E., Coules H.E., Wu G., Khan R.H.U., Xu X., Williams S. (2019). Wire + Arc Additively Manufactured Inconel 718: Effect of post-deposition heat treatments on microstructure and tensile properties. Mater. Des..

[88] Salvi H., Vesuwala H., Raval P., Badheka V., Khanna N. (2023). Sustainability analysis of additive + subtractive manufacturing processes for Inconel 625. Sustain. Mater. Technol..

[89] Sharifitabar M., Khorshahian S., Shafiee Afarani M., Kumar P., Jain N.K. (2022). High-temperature oxidation performance of Inconel 625 superalloy fabricated by wire arc additive manufacturing. Corros. Sci..

[90] Raghupatruni P., Kumar S.A., Salunkhe S., Amancio-Filho S.T., Davim J.P. (2023). Chapter 4—Review of Microstructure and Mechanical properties of materials manufactured by direct energy deposition. Advances in Metal Additive Manufacturing.

[91] Teixeira Ó., Silva F.J.G., Atzeni E. (2021). Residual stresses and heat treatments of Inconel 718 parts manufactured via metal laser beam powder bed fusion: An overview. Int. J. Adv. Manuf. Technol..

[92] Pérez-Ruiz J.D., De Lacalle L.N.L., Urbikain G., Pereira O., Martínez S., Bris J. (2021). On the relationship between cutting forces and anisotropy features in the milling of LPBF Inconel 718 for near net shape parts. Int. J. Mach. Tools Manuf..

[93] Soffel F., Eisenbarth D., Hosseini E., Wegener K. (2021). Interface strength and mechanical properties of Inconel 718 processed sequentially by casting, milling, and direct metal deposition. J. Mater. Process. Technol..

[94] Arlyapov A., Volkov S., Promakhov V., Matveev A., Babaev A., Vorozhtsov A., Zhukov A. (2022). Study of the Machinability of an Inconel 625 Composite with Added NiTi-TiB2 Fabricated by Direct Laser Deposition. Metals.

[95] Danish M., Aslantas K., Hascelik A., Rubaiee S., Gupta M.K., Yildirim M.B., Ahmed A., Mahfouz A.B. (2022). An experimental investigations on effects of cooling/lubrication conditions in micro milling of additively manufactured Inconel 718. Tribol. Int..

[96] Ceritbinmez F., Günen A., Gürol U., Çam G. (2023). A comparative study on drillability of Inconel 625 alloy fabricated by wire arc additive manufacturing. J. Manuf. Process..

[97] Stachowiak A., Wieczorek D., Gruber K., Bartkowski D., Bartkowska A., Ulbrich D. (2023). Comparison of tribocorrosion resistance of Inconel® 718 alloy manufactured by conventional method and laser powder bed fusion method. Tribol. Int..

[98] Shipway P.H., Liskiewicz T., Dini D. (2023). The role of tribologically transformed structures and debris in fretting of metals. Fretting Wear and Fretting Fatigue.

[99] Li K., Ma R., Zhang M., Chen W., Li X., Zhang D.Z., Tang Q., Murr L.E., Li J., Cao H. (2022). Hybrid post-processing effects of magnetic abrasive finishing and heat treatment on surface integrity and mechanical properties of additively manufactured Inconel 718 superalloys. J. Mater. Sci. Technol..

[100] Gürgen S., Sofuoğlu M.A., Gupta K., Pramanik A. (2021). Chapter 4—Advancements in conventional machining: A case of vibration and heat-assisted machining of aerospace alloys. Advanced Machining and Finishing.

[101] Vignesh M., Ramanujam R., Gupta K., Paulo Davim J. (2020). Chapter 9—Laser-assisted high speed machining of Inconel 718 alloy. High Speed Machining.

[102] Venkatesan K. (2017). The study on force, surface integrity, tool life and chip on laser assisted machining of inconel 718 using Nd:YAG laser source. J. Adv. Res..

[103] Parida A.K., Maity K. (2018). Comparison the machinability of Inconel 718, Inconel 625 and Monel 400 in hot turning operation. Eng. Sci. Technol. Int. J..

[104] Baek J.-T., Woo W.-S., Lee C.-M. (2018). A study on the machining characteristics of induction and laser-induction assisted machining of AISI 1045 steel and Inconel 718. J. Manuf. Process..

[105] Choi Y.H., Lee C.M. (2018). A study on the machining characteristics of AISI 1045 steel and inconel 718 with circular cone shape in induction assisted machining. J. Manuf. Process..

[106] Moon S.-H., Lee C.-M. (2018). A study on the machining characteristics using plasma assisted machining of AISI 1045 steel and Inconel 718. Int. J. Mech. Sci..

[107] Kim E.J., Lee C.M. (2019). A Study on the Optimal Machining Parameters of the Induction Assisted Milling with Inconel 718. Materials.

[108] Baptista A., Silva F., Porteiro J., Míguez J., Pinto G. (2018). Sputtering Physical Vapour Deposition (PVD) Coatings: A Critical Review on Process Improvement and Market Trend Demands. Coatings.

[109] Sousa V.F.C., Da Silva F.J.G., Pinto G.F., Baptista A., Alexandre R. (2021). Characteristics and Wear Mechanisms of TiAlN-Based Coatings for Machining Applications: A Comprehensive Review. Metals.

[110] Kim E.-J., Lee C.-M. (2020). Experimental study on power consumption of laser and induction assisted machining with inconel 718. J. Manuf. Process..

[111] Jeong H.-I., Lee C.-M. (2021). A study on improvement of tool life using a heat shield in laser assisted machining to Inconel 718. Opt. Laser Technol..

[112] Liu S., Xiao G., Lin O., He Y., Song S. (2023). A new one-step approach for the fabrication of microgrooves on Inconel 718 surface with microporous structure and nanoparticles having ultrahigh adhesion and anisotropic wettability: Laser belt processing. Appl. Surf. Sci..

[113] Zhang H., Deng B., Lin J., Tang X., Yan R., Peng F. (2023). Spatial gradient prediction and characterization of yield strength in the heat-affected zone in laser-assisted machining of Inconel 718. Opt. Laser Technol..

[114] Vipindas K., Kuriachen B., Mathew J., Gupta K., Pramanik A. (2021). Chapter 13—An insight on ultrasonic machining technology. Advanced Machining and Finishing.

[115] Airao J., Nirala C.K., Khanna N. (2022). Novel use of ultrasonic-assisted turning in conjunction with cryogenic and lubrication techniques to analyze the machinability of Inconel 718. J. Manuf. Process..

[116] Zhang X., Peng Z., Liu L., Zhang X. (2022). A Tool Life Prediction Model Based on Taylor′s Equation for High-Speed Ultrasonic Vibration Cutting Ti and Ni Alloys. Coatings.

[117] Zhang M., Zhang D., Guo H., Gao Z., Geng D., Liu J., Jiang X. (2020). High-Speed Rotary Ultrasonic Elliptical Milling of Ti-6Al-4V Using High-Pressure Coolant. Metals.

[118] Peng Z., Zhang X., Zhang D. (2021). Integration of finishing and surface treatment of Inconel 718 alloy using high-speed ultrasonic vibration cutting. Surf. Coat. Technol..

[119] Yin X., Liu Y., Zhao S., Li X., Geng D., Zhang D. (2023). Tool wear and its effect on the surface integrity and fatigue behavior in high-speed ultrasonic peening milling of Inconel 718. Tribol. Int..

[120] Yin X., Li X., Liu Y., Geng D., Zhang D. (2023). Surface integrity and fatigue life of Inconel 718 by ultrasonic peening milling. J. Mater. Res. Technol..

[121] Sobih M., Gupta K., Pramanik A. (2021). Chapter 12—Laser-based machining – an advanced manufacturing technique for precision cutting. Advanced Machining and Finishing.

[122] Ahmed N., Rafaqat M., Pervaiz S., Umer U., Alkhalefa H., Shar M.A., Mian S.H. (2019). Controlling the material removal and roughness of Inconel 718 in laser machining. Mater. Manuf. Process..

[123] Singh H., Bhoi N.K., Jain P.K., Gupta K., Pramanik A. (2021). Chapter 6—Developments in abrasive water jet machining process—From 1980 to 2020. Advanced Machining and Finishing.

[124] Alsoruji G., Muthuramalingam T., Moustafa E.B., Elsheikh A. (2022). Investigation and TGRA based optimization of laser beam drilling process during machining of Nickel Inconel 718 alloy. J. Mater. Res. Technol..

[125] Pan Z., Feng Y., Hung T.-P., Jiang Y.-C., Hsu F.-C., Wu L.-T., Lin C.-F., Lu Y.-C., Liang S.Y. (2017). Heat affected zone in the laser-assisted milling of Inconel 718. J. Manuf. Process..

[126] Haghbin N., Spelt J.K., Papini M. (2015). Abrasive waterjet micro-machining of channels in metals: Comparison between machining in air and submerged in water. Int. J. Mach. Tools Manuf..

[127] Venkateshwar Reddy P., Suresh Kumar G., Satish Kumar V. (2020). Multi-response Optimization in Machining Inconel-625 by Abrasive Water Jet Machining Process Using WASPAS and MOORA. Arab. J. Sci. Eng..

[128] Salinas L.C., Moussaoui K., Hejjaji A., Salem M., Hor A., Zitoune R. (2021). Influence of abrasive water jet parameters on the surface integrity of Inconel 718. Int. J. Adv. Manuf. Technol..

[129] Srirangarajalu N., Vijayakumar R., Rajesh M. (2022). Multi performance investigation of Inconel-625 by abrasive aqua jet cutting. Mater. Manuf. Process..

[130] Vijayakumar R., Srirangarajalu N., Santhanakumar M., Paul N.E.E., Rajesh M. (2023). Investigation of Abrasive Aqua Jet Hole Making (AAJHM) parameters using desirability analysis on Inconel-625 space alloy. J. Manuf. Process..

[131] Barman A., Das M., Gupta K., Pramanik A. (2021). Chapter 17—Fundamental understanding and latest developments in magnetic field assisted finishing processes. Advanced Machining and Finishing.

[132] Liu J., Zou Y. (2023). Study on Elucidation of the Roundness Improvement Mechanism of the Internal Magnetic Abrasive Finishing Process Using a Magnetic Machining Tool. J. Manuf. Mater. Process..

[133] Wang B., Ji R., Gong Z., Zhao Q., Liu Y., Jin H., Wang L., Xu Z., Cai B., Ma J. (2022). Effect of water in oil emulsion on the surface quality of Inconel 718 alloy during coupling electrical pulse and ultrasonic treatment. Surf. Coat. Technol..

[134] Li S., Xiao G., Zhuo X., Chen B., Zhao Z., Huang Y. (2023). Fatigue performance and failure mechanism of ultrasonic-assisted abrasive-belt-ground Inconel 718. Int. J. Fatigue.

[135] Xie H.-L., Li J.-R., Liao Z.-Y., Wang Q.-H., Zhou X.-F. (2020). A robotic belt grinding approach based on easy-to-grind region partitioning. J. Manuf. Process..

[136] Song K., Xiao G., Chen S., Liu X., Huang Y. (2023). A new force-depth model for robotic abrasive belt grinding and confirmation by grinding of the Inconel 718 alloy. Robot. Comput. -Integr. Manuf..

[137] Zhao Y., Ratay J., Li K., Yamaguchi H., Xiong W. (2022). Effects of Magnetic Abrasive Finishing on Microstructure and Mechanical Properties of Inconel 718 Processed by Laser Powder Bed Fusion. J. Manuf. Mater. Process..

